# Quantification and Analysis of Micro-Level Activities Data from Children Aged 1–12 Years Old for Use in the Assessments of Exposure to Recycled Tire on Turf and Playgrounds

**DOI:** 10.3390/ijerph19042483

**Published:** 2022-02-21

**Authors:** Nicolas Lopez-Galvez, Jocelyn Claude, Patty Wong, Asa Bradman, Carly Hyland, Rosemary Castorina, Robert A. Canales, Dean Billheimer, Elmira Torabzadeh, James O. Leckie, Paloma I. Beamer

**Affiliations:** 1 Mel and Enid Zuckerman College of Public Health, University of Arizona, Tucson, AZ 85724, USA; dean.billheimer@arizona.edu (D.B.); pbeamer@arizona.edu (P.I.B.); 2 San Diego State University Research Foundation, School of Public Health, San Diego, CA 92182, USA; 3 Office of Environmental Health Hazard Assessment (OEHHA), California Environmental Protection Agency (EPA), Sacramento, CA 95812, USA; jocelyn.claude@oehha.ca.gov (J.C.); patty.wong@oehha.ca.gov (P.W.); 4 Center for Environmental Research and Children’s Health, University of California, Berkeley, CA 94704, USA; abradman@berkeley.edu (A.B.); carlybarker@berkeley.edu (C.H.); rcastori@berkeley.edu (R.C.); 5 Department of Public Health, School of Social Sciences, Humanities, and Arts, University of California, Merced, CA 95343, USA; 6 Department of Environmental and Occupational Health, Milken Institute School of Public Health, George Washington University, Washington, DC 20052, USA; rcanales@email.gwu.edu; 7 Center for Biomedical Informatics and Biostatistics, University of Arizona, Tucson, AZ 85724, USA; etorabzadeh@gmail.com; 8 Department of Civil and Environmental Engineering, Stanford University, Stanford, CA 94305, USA; leckie@stanford.edu

**Keywords:** micro-level activity time series, artificial turf, playgrounds, activity patterns, recycled tire crumb rubber

## Abstract

Background: There are growing health concerns about exposure to toxicants released from recycled tire rubber, which is commonly used in synthetic turf and playground mats. To better estimate children’s exposure and risk from recycled tire rubber used in synthetic turf and playground mats, there is a need to collect detailed accurate information on mouthing activity and dermal contact behaviors. The objective of this study was to quantify and analyze micro-level activity time series (MLATS) data from children aged 1–12 years old while playing (non-sport-related games) at turf-like locations and playgrounds. Another objective was to estimate the incidental ingestion rate of rubber crumb among children. Methods: Hand and mouth contact frequency, hourly duration, and median contact duration with different objects were calculated for children playing on turf (i.e., parks, lawns, and gardens) (n = 56) and for children playing on playground structures (n = 24). Statistically significant differences between males and females as well as children’s age groups were evaluated. The daily incidental ingestion rate of rubber crumb was calculated. Results: For children playing on turf, there were significant differences between younger (1–6 y) and older (7–12 y) children for the mouthing median duration with non-dietary objects and all objects. For children playing on playground structures, we found significant mouthing frequency differences between younger (1–6 y) and older children (7–12 y) with all objects, and for mouthing median duration with non-dietary objects. There were no significant differences between males and females playing on artificial turf-like surfaces or playground mats. Our estimated mean incidental ingestion rate was 0.08, 0.07, and 0.08 g rubber crumb/day for children <2, 2–6, and 6–11 years old, respectively. Discussion: our results suggest that age and contact duration should be considered in risk assessment models to evaluate mouthing activities when children are playing on artificial turf surfaces or playground mats.

## 1. Introduction

The use of synthetic turf in sport fields, parks, yards, and playgrounds has gained popularity in recent years due to the low water and maintenance requirements. Most synthetic turf-like materials in sports fields share the same basic infill composition, which is made of recycled tire crumb rubber [1,2]. In addition, because of its cushioning characteristics, poured-in-place (PIP) rubber surface systems have been used more frequently worldwide in private and public playground mats over the last 10 years, replacing natural turf on playgrounds [3]. There have been increasing public health concerns raised about the safety of using recycled tire rubber (i.e., crumb rubber) as infill in synthetic turf and PIP rubber surfaces due to the potential human exposure to recycled-rubber-derived chemicals and associated negative health effects [4,5]. Previous studies have detected several potentially harmful chemicals in this recycled tire crumb rubber including volatile organic compounds (VOCs), semi-volatile organic compounds (SVOCs), polycyclic aromatic hydrocarbons (PAHs), and heavy metals such as lead and zinc [1,6]. Previous artificial turf exposure studies have primarily focused on children playing sports, and data gaps exist regarding other related exposures such as among children watching sport events (i.e., child spectators or bystanders), children engaging in non-sporting activities on landscape synthetic grass, and PIP rubber surface playgrounds or rubber mulch nuggets made from recycled tires at playgrounds or homes [4,5].

Exposure to chemicals and metals derived from crumb rubber utilized as an infill material may occur via inhalation, dermal contact, and/or ingestion. In terms of inhalation, some VOCs and SVOCs can be released and inhaled from crumb rubber, especially in higher temperatures making these chemicals more accessible to children playing on these surfaces [7]. In addition, due to the fact of their activity patterns and physiological characteristics, children may have increased exposure and be more susceptible to environmental toxicants, including these rubber-derived chemicals, than adults [8,9,10,11]. For example, children spend more time crawling on the ground, where they may come into contact with chemicals, have greater hand-to-mouth contact, and are undergoing rapid periods of physiologic development [12]. Hence, it is crucial to evaluate the exposure to artificial turf and PIP rubber surfaces by characterizing children’s dermal contact with these materials and their mouthing behaviors.

The collection and use of micro-activity data are fundamental in the accuracy of models that assess exposure to environmental contaminants including artificial turf and recycled rubber contaminants. Mouthing and hand contact activities are key parameters to evaluate non-dietary ingestion and dermal exposure to chemicals [13], and videotaping is considered the most accurate method for collecting micro-activity patterns to characterize these exposures [8,14]. Previous studies have quantified mouthing and hand contact behaviors in children playing outdoors [10,11,12,13,15,16,17,18]; however, only two of those studies reported data on children over 6 years of age [13,18]. No previous studies have specifically quantified the mouthing and dermal contact behavior of children playing outside on turf and playground structures.

The objective of this study was to quantify dermal and mouthing contact behaviors by analyzing recorded videotapes and pre-existing micro-level activity time series (MLATS) data of children ages 1–12 years old playing outdoors on turf and playground structures. While previous studies have evaluated micro-activity data from videotapes of children playing outdoors, this study will help to better understand how children may interact with artificial turf and playground mats [10,11,14,15,19], information that is necessary to examine potential exposure to contaminants from artificial turf, playground mats, and rubber mulch nuggets under playgrounds. This study expands on the analysis of available MLATS data on children engaging in non-sporting activities, who could be exposed to toxicants released from recycled tire crumb rubber while playing outside on playground mats and artificial turf-like materials. Additionally, to demonstrate the impact and usefulness of these data, we estimated the exposure to non-dietary or incidental ingestion of crumb rubber using current recorded MLATS data.

## 2. Materials and Methods

### 2.1. Data Collection

Children MLATS data and videotapes were collected from two previous studies, the Outdoor Residential Exposure Task Force (ORETF) project and the US Environmental Protection Agency (EPA) study based at Stanford University from the years 1998–2000. Details on the methods have been previously described [11,13,15,16,20]. Briefly, videotaped activities were transcribed into computer text files using the Virtual Timing Device (VTD) developed by the Stanford Exposure Research Group. The VTD palette used for translation of micro-level activity data in this study is presented in detail in AuYeung et al. [15]. This palette allowed the translator to record the child’s location, contact type, and object/surface type. Human subject IRB approval was obtained (Protocol number: 1810983273).

For the ORTEF project, MLATS data were collected for 15 body parts, including hands and mouth, by videotaping 36 children aged 1–12 years while they were playing outdoors. Meanwhile, for the EPA study, MLATS data were collected for mouth and hands by videotaping 20 children aged 1–6 years while they were playing outdoors. For both studies, children were videotaped for approximately 2 h each. We used these preexisting data and videos to quantify and summarize the mouthing and dermal contact behavior of 56 children while playing only on turf and playgrounds. It is important to mention that most of our MLATS data were collected from children playing on natural turf/grass, and we assumed that similar activity patterns would be observed among children playing on artificial turf.

### 2.2. MLATS Data Processing

To characterize “*children playing on turf*”, the translated plain text files, obtained from the previous studies, for all 56 children for the right hand, left hand, and mouthing activities were used for analysis. The objects/surfaces were selected and combined as presented in Table 1. Participants’ contacts while playing in the location categories of yard, garden, and park were extracted and grouped as being one turf-like location using RStudio V1.1 [21]. Videotapes were reviewed to confirm turf-like locations.

To characterize “*children playing on playgrounds*”, additional videography analysis was conducted. Since “playground” was not categorized in the previous studies, all of the existing videotapes (n = 56) were re-viewed to determine footage where a playground structure was observed. The exact footage time and duration when children played on or near playground structures was recorded and then a specific “playground” location was added to each corresponding MLATS file. The objects/surfaces selected for analysis were the same as for turf and are presented in Table 1. Since there are many different types of playground flooring surfaces, all floor types were grouped into one category for the playground sub-locations, because it is fundamental to learn children’s micro-activity while playing in a playground environment (Table 1).

### 2.3. Data Analysis

Using RStudio V1.1, right hand, left hand, and mouth contact frequency; hourly contact duration; median contact duration with the selected objects/surface categories (Table 1) were calculated for each child while playing on turf and playground structures, respectively. Contact frequency (i.e., events/h) was calculated by summing the total number of contact events by hands or mouth with turf, floor, or any specific object category divided by the total time that the child was in view. Hourly contact duration (i.e., min/h) was calculated by summing the total time in minutes that hands or mouth were in contact with turf, floor, or any specific object category divided by the total time in view (i.e., hours). Median duration (i.e., seconds) was obtained from the contact duration for the hands or mouth contact events with turf, floor, or any specific object/category during the time in view for each child. It was the median value of all contacts of the body part (mouth/hands) with the turf, floor, or object.

The Wilcoxon signed rank test was used to assess differences in the activity variables (i.e., contact frequency, hourly duration, and median duration) between the right and left hands. If no differences were observed, then data for the hands were combined and reported as “both hands”. To determine differences between males and females, a two-sided Wilcoxon rank-sum test was utilized. The Kruskal–Wallis test was used to assess if there were differences across age groups (i.e., 1 to <2; 2 to <3; 3 to <6; 6 to <11; 1–16 y) recommended by the US EPA, 2005 [26]. To determine if activities correlated with age, Spearman’s rank correlation coefficient was computed. In addition, differences between the activities of 1–6 year old children and 7–12 year old children were assessed using the two-sided Wilcoxon rank-sum tests. All of the statistical tests were conducted using STATA V13.0. [27].

### 2.4. Estimation of the Daily Incidental Crumb Rubber Ingestion

We used the following equations to estimate the daily incidental ingestion of crumb rubber:

(1) $$IR_{{daily}_{ing}}=\left(IR_{indirect}\times AET\right)\times CF1$$

(2) $$IR_{indirect}=IR_{HTM}+IR_{OTM}+IR_{HTOTM}$$

(3) $$IR_{HTM}=AF_{hand}\times SA_{HTM}\times TF_{HTM}\times f_{HTM}\times CF2$$

(4) $$IR_{OTM}=AF_{obj}\times SA_{obj}\times TF_{obj}\times f_{OTM}\times CF2$$

(5) $$IR_{HTOTM}=AF_{hand}\times SA_{indirect}\times TF_{indirect}\times f_{HTOTM}\times CF2$$

where:

*IR_daily_ing_* = daily average of the total amount of crumb rubber ingested over a year for a specific receptor category and age group (g_crumb rubber_/day);
*IR_indirec_*_t_ = amount of crumb rubber ingested via all the indirect ingestion pathways during an event for a specific receptor category and age group (g_crumb rubber_/h);
*IR_HTM_* = amount of crumb rubber indirectly ingested via hand-to-mouth (HTM) behaviors during an event for a specific receptor category and age group (g_crumb rubber_/h);
*IR_OTM_* = amount of crumb rubber indirectly ingested via the object-to-mouth (OTM) behaviors during an event for a specific receptor category and age group (g_crumb rubber_/h);
*IR_HTOTM_* = amount of crumb rubber indirectly ingested via the hand-to-object-to-mouth (HTOTM) behaviors during an event for a specific receptor category and age group (g_crumb rubber_/h);
*AET* = annual event time or number of hours that children spent playing on a grass field for a year for a specific age group and receptor category (h/year);
*AF_hand_* = adherence factor: the amount of crumb rubber that adheres to the skin per unit of surface area of the hand (mg_crumb rubber_/cm^2^ per contact);
*AF_obj_* = adherence factor of crumb rubber for an object (mg_crumb rubber_/cm^2^ per contact);
*SA_HTM_* = surface area of the part of the hand in direct contact with the mouth (cm^2^);
*SA_indirect_* = surface area of the part of the hand in contact with the object reaching the mouth (cm^2^);
*SA_obj_* = surface area of the part of the object reaching the mouth (cm^2^);
*TF_HTM_* = fraction of the crumb rubber transferred from the part of the hand in contact with the mouth to the mouth (unitless);
*TF_obj_* = fraction of the amount of crumb rubber transferred from the object into the mouth (unitless);
*TF_indirect_* = fraction of the amount of crumb rubber transferred from the hand to an object then into the mouth (unitless);
*CF*1 = conversion factor (1 year/365 days);
*CF*2 = conversion factor (0.001 g_crumb rubber_/mg_crumb rubber_);
*f_HTM_* = frequency of HTM contacts, i.e., the number of hand touches to the mouth or peri-buccal area per hour during an event (contacts/h);
*f_OTM_* = frequency of OTM contacts (contacts/h);
*f_HTOTM_* = frequency of HTOTM contacts in an hour (contacts/h).

Contact frequencies: The frequency of hand-to-mouth (*f_HTM_*), object-to-mouth (*f_OTM_*), and hand-to-object-to-mouth (*f_HTOTM_*) behaviors were summarized from the collected children MTLATS data to calculate the indirect ingestion of crumb rubber. The contact frequencies of recorded children playing on turf were used for this calculation. The mean, median, and 95th percentile values of the collected contact frequencies were used to derive the corresponding mean, median, and 95th percentile values of the daily indirect ingestion using Equations (2)–(5).

Annual event time (AET): corresponds to the number of hours that children spent playing on a grass field for a year, which was converted from minutes per day to h/year by multiplying conversion factors for 365 days and 1/60 min (Table 1).

Adherence factors: The value for the crumb rubber adherence factor for the hand, *AF_hand_*, was estimated to be 0.026 mg_crumb rubber_/cm^2^ per contact [22]. We assumed that adherence was uniform across the surface of the hand, that crumb rubber loading on the hand reached a steady level after several contacts, and that the rate of transfer from subsequent hand-to-object contacts was less than or equal to the field-to-hand loading rate [28]. The adherence factor of crumb rubber for an object, *AF_obj_*, describes the amount of crumb rubber that adheres to an object after contact with the playground floor or PIP rubber surfaces. We did not measure any adherence factors of crumb rubber to objects, but we anticipated toys and pacifiers to be the most likely objects in OTM behaviors. These objects are often made of materials such as plastics or silicone, which have adherence properties similar to and act in a manner similar to the skin [29,30,31]. It is for this reason that some exposure studies used wristbands made of silicone to monitor or simulate personal exposures to environmental chemicals [32]. In the absence of data on the adherence of crumb rubber to objects, we adopted the literature value of 0.026 mg/cm^2^ per contact for *AF_hand_* measured by Kissel and coworkers (1996) [22] as the value of *AF_obj_*. We assumed that the material of the objects on the field acted in a similar way to the skin. The crumb rubber adherence factors are described in Table 1.

Surface Areas: The skin surface area, *SA_HTM_*, represents the surface area of the part of the hand in direct contact with the mouth. For this study, each hand contact with the mouth was assumed to be four fingers of the grasping side (the palm side) of one hand. Following the Office of Environmental Health Hazard Assessment (OEHHA) guidelines, the grasping side of one hand was assumed to represent 25% of the total surface area of both hands [23]. Each finger represents ten percent of the grasping side surface of one hand, so four fingers were assumed to represent 10% of total surface area of both hands. To obtain *SA_HTM_*, the *SA_hands_* is multiplied by 0.10. For this study, the surface area of the part of the object reaching the mouth, *SA_obj_*, was assumed to be limited by the surface area around the mouth area of the face. These parameter values were age and gender specific. The face was assumed to be one-third of the surface area of the head [25], and we assumed the area around the mouth to be the lower one-third of the face. To obtain *SA_obj_*, the head surface area, *SA_head_*, was multiplied by 1/3. With respect to the contact of indirect surface area, *SA_indirect_*, the part of the hand in direct contact with an object varies based on the type of HTOTM contact and the receptor category. We used the collected MLATS data to determine the type of objects and the part of the hand involved in the HTOTM contacts. We used the surface area of the hand (*SA_hands_*) to estimate the area portion in contact with an object. We assumed that contact would be with the whole hand if eating one-handed or only the fingers if eating with two hands or a few fingers if using a water bottle. These types of contact represented 25% and 12.5%, respectively, of the surface area of the hand. As a conservative estimate, one hand was assumed to be used when eating food or drinking while playing, i.e., 25% of the surface area of both hands. For non-dietary objects, such as toys and pacifiers, it was also assumed that one hand contacts the object. The surface area values are provided in Table 1.

Transfer Fractions: The hand-to-mouth transfer factor, TF_HTM_, is a unitless factor describing the fraction of the amount of crumb rubber that is transferred from the portion of the skin of the hand in contact with the mouth. We assumed this factor to be independent to age and receptor category. We adopted a value of 0.5 (unitless) according to the OEHHA guidelines [23,24] for the transfer of lead from consumer products. This value was derived from a study [33] of the removal of 3 pesticides (i.e., chlorpyrifos, pyrethrin I, and piperonyl butoxide) from the hands of three human subjects with human saliva, artificial saliva, and dioctyl sodium sulfosuccinate (DSS, a mild surfactant)-moistened wipes. The OTM transfer factor, *TF_obj_*, is the fraction of the amount of crumb rubber transferred from the surface of an object into the mouth in an OTM incident. We also anticipated this factor to be the same regardless of the age and activity category of the field users. We assumed that the objects used for OTM activity were made of materials that had similar properties as the skin. Hence, we assumed that each OTM contact would transfer 50% of the crumb rubber adhered to the surface of the objects to the mouth, similar to TF_HTM_. The HTOTM transfer factor, *TF_indirect_*, is a unitless factor describing the fraction of the amount of crumb rubber that is transferred from the part of the hand in contact with an object to the object and then into the mouth. This factor is independent of age or activity category of field users.

(6) $$TF_{indirect}=TF_{direct}\times \left(1-TF_{loss}\right)$$

where:

$TF_{indirect}$ = fraction of the amount of crumb rubber transferred from the portion of a hand in contact with an object to the mouth (unitless);

$TF_{direct}$ = fraction of the amount of crumb rubber transferred from the hand to the mouth (unitless);

$TF_{loss}$ = the fraction of crumb rubber lost from the hand prior to transfer into the mouth (unitless).

The loss occurs after certain activities, such as hand washing or wiping hands on clothing, prior to transfer onto the object which eventually transfers into the mouth. It accounts for the possibility of multiple steps between hand loading and transfer to the mouth, for example, when an athlete wipes their hands on their shirt and then picks up a piece of fruit to eat. Hence, following OEHHA guidelines [23,24], a value of 50% for both *TF_direct_* and *TF_loss_* was used. We applied a *TF_loss_* value of 0% for the synthetic turf study considering the low probabilities of handwashing at the same time children play. The resulting value for *TF_indirect_* used for our calculation was 0.25. All transfer factors used in this study are presented in Table 1.

## 3. Results

A total of 43 h (2548 min) of footage was collected from the 56 children with a median footage time per child of 112 min (range: 60–133 min). This was the total amount of footage available for the analysis. As presented in Table 2, a total of 1812 min (71%) of the time in view were of children playing on turf and 531 min (21%) occurred while they played on playgrounds. The footage not in view of children playing on turf (3.9% of total time) and playgrounds (1.5% of total time) was excluded from analysis (Table 2). As shown in Table 2, there were comparable numbers of male and female participants playing on turf and playgrounds. The highest number of children playing on turf were between the ages of 6 and <11 years, whereas the highest number of children playing on playgrounds were between 3 and <6 years old. Only six children (11%) aged 11 years or older were recorded playing on turf, while no children 11 years or older were recorded playing on playgrounds (Table 2).

### 3.1. MLATS of Children Playing on Turf

Since there were no significant differences in the contact frequency, hourly duration, and median duration with objects and surfaces between the right and left hands (Tables S1–S9), both hands were combined and summarized as shown in Table 3. The median grass contact frequency for both hands combined was 4.1 events/h and a maximum contact frequency of 251.8 events/h. The median hourly contact duration for both hands with grass was 0.2 min/h and a maximum value of 12.0 min/h. The median of the median contact duration of both hands combined with grass was 2.0 s, and one child had a median contact duration of both hands with grass of 13.0 s (Table 3). 

**Figure 1:**
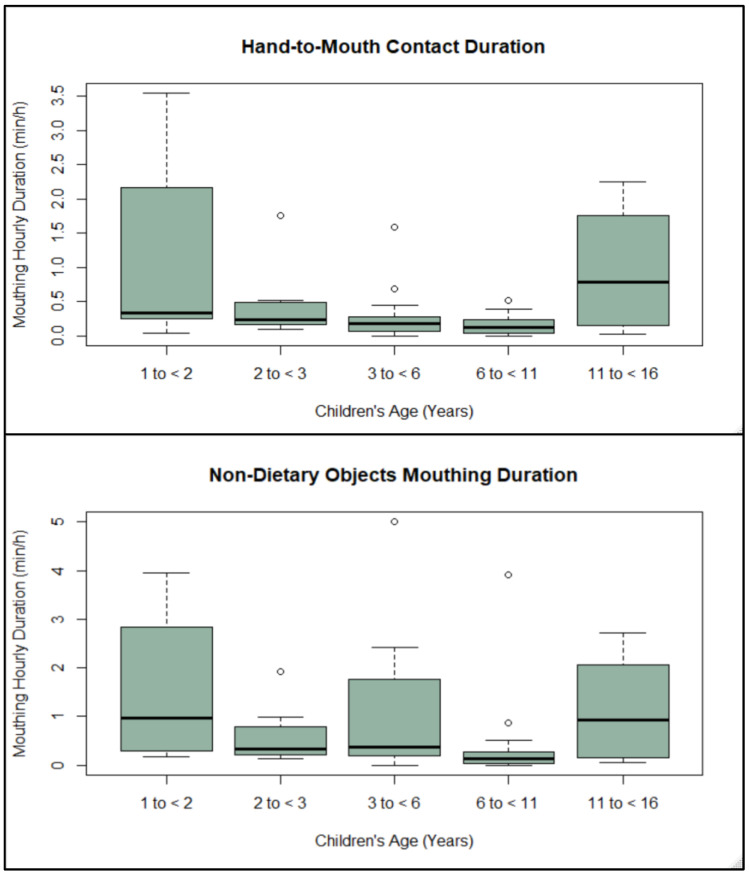
Mouthing hourly duration (min/h) with non-dietary objects and hands while playing on turf by age groups.

There were no significant correlations between age and both hand activities and no significant differences in contact frequency, hourly contact duration, or median contact duration across the EPA age groups or by gender while playing on turf (Tables S10–S15). Additionally, there were no significant differences between children aged 1–6 and 7–12 years for both hands contact activities (Table S12A–C in the Supplementary Materials). Among all participants, the median mouthing frequency for grass was 0.0 events/h, and the maximum was 2.5 events/h (Table 3). The median mouthing frequencies were 7.6 and 10.9 events/h for hands and non-dietary objects, respectively. The median mouthing hourly contact duration for grass was 0.0 min/h and the maximum was 0.1 min/h. The median mouthing hourly contact durations were 0.2 and 0.3 min/h for hands and non-dietary objects, respectively. The median of mouthing median contact duration for grass was 1.0 s, and the maximum was 2.5 s. The median of mouthing median contact durations was 1.0 s for both hands and non-dietary objects (Table 3). These contacts could indicate additional exposure to crumb rubber if there have been transfers to the child’s hands or the objects that they are placing in their mouths. Although age was not significantly correlated with mouthing contact frequencies, age was negatively correlated (*p* < 0.05) with the hourly contact duration of mouthing activities for non-dietary objects and all objects. Age was also negatively correlated (*p* < 0.05) with the median contact duration of mouthing activities for hands and non-dietary objects (Table S16). As presented in Figure 1, there were significant differences across the EPA-recommended age groups for mouthing hourly contact duration with hands and non-dietary objects. 

Children below the age of 3 years and children older than 11 years of age had significantly higher mouthing hourly contact duration with hands and non-dietary objects than the other age groups (Figure 1, Table S18). There were no significant differences in mouthing contact frequency and contact median duration among EPA age groups. However, there were significant differences between younger and older children (1–6 years old vs. 7–12 years old) in mouthing median duration with non-dietary objects (Figure S1, Table S20). There were no significant differences in contact frequency, hourly contact duration, or median contact duration by gender (Tables S21–S23).

### 3.2. MLATS of Children Playing on Playground

Because there were no significant differences in the contact frequency, hourly contact duration, and median contact duration with objects and surfaces between the right and left hands while children were playing on playgrounds (Tables S24–S32), both hands were combined and summarized in Table 4. The median contact frequency with ground surfaces (i.e., playground mats) for both hands combined was 12.1 events/h. The median hourly hands contact duration with ground surfaces was 0.6 min/h, while the maximum value was 10.1 min/h. The median value of the median duration for contact of both hands with floors was 2.0 s (Table 4). As shown in Table S33 in the Supplementary Materials, age was not significantly correlated with both hands’ contact activities. There were neither statistically significant differences among EPA age groups nor significant differences between younger and older children (aged 1–6 years old and 7–12 years old) for contact activities with both hands. In addition, for both hands’ contact activities, there were no significant differences in frequency, hourly contact duration, or median contact duration by gender (Tables S34–S36).

Regarding mouthing activity, the mouthing contact frequency, hourly mouthing duration, and median mouthing duration for children playing on playgrounds were summarized in Table 4.

Only one child (1/24) contacted “floors” with the mouth, resulting in a contact frequency of 2.3 events/h. The median mouthing frequencies were 9.8 and 10.2 events/h for hands and non-dietary objects, respectively. The hourly mouthing duration for the one child who contacted “floors” with their mouth was 0.1 min/h. The median hourly mouthing duration was 0.1 and 0.0 min/h for hands and non-dietary objects, respectively. The mouthing duration for the one child that contacted “floors” with the mouth was 1.0 s. The median of the mouthing median contact durations was 1.0 s for hands and non-dietary objects, respectively (Table 4). These mouthing contacts could indicate additional exposure to playground mat chemicals that have transferred to the children’s hands or the objects that they are placing in their mouths. Age was negatively correlated (*p* < 0.05) with the contact frequency, hourly mouthing duration, and median duration of mouthing activities with total objects. In addition, age was negatively correlated with hourly mouthing duration and median mouthing duration for non-dietary objects. Finally, age was negatively correlated (*p* < 0.05) with mouthing frequency and hourly mouthing duration for both hands (Table S37). There were significant differences (*p* < 0.05) among the EPA age groups with respect to mouthing frequency for non-dietary objects and all objects. Similarly, a significant difference was observed for the mouthing hourly duration for non-dietary objects (Tables S38–S40). There were also significant differences between younger (1–6 years old) and older children (7–12 years old) groups for the mouthing frequency and hourly duration with all objects and for the mouthing hourly duration with non-dietary objects (Tables S41 and S42). In relation to gender, there were no significant differences in mouthing contact frequency or mouthing hourly contact duration by gender. However, girls had significantly longer median mouthing durations with all objects while playing on playgrounds (Table S44).

For both hand contacts with grass in turf or floors in playgrounds, the median frequency and median hourly contact durations were higher when children played at playgrounds (12.1 events per hour and 0.6 min/h, respectively) than when children played on turf (4.1 events per hour and 0.2 min/h, respectively). In addition, for both hands contact activity when children played on turf or playground structures, there were neither significant differences nor correlations with children’s age or gender.

### 3.3. Daily Incidental Crumb Rubber Ingestion

As presented in Table 5, the estimated daily incidental crumb rubber ingestion among children was similar across all ages with mean values of 0.08 g_crumb rubber_/day, 0.07 g_crumb rubber_/day, and 0.08 g_crumb rubber_/day for children under 2 years of age, between 2 and 6 years of age, and children between 6 and 11 years of age, respectively.

## 4. Discussion

Although while playing on turf-like surfaces, older children touched grass and non-dietary objects with their hands more frequently and for longer durations than younger children, which is contrary to results previously reported [13,15], these differences were not significant. On the other hand, there was a decrease in hand contact behaviors as a function of age when children played on playgrounds. Younger children (under 7 years of age) had more frequent and longer contacts of hands with the floors and non-dietary objects than older children (7–12 years of age) while playing on playground structures. This decrease could be related to human developmental stages, as children become older, they are less likely to continue playing on playgrounds regardless of their gender [34], but when older children played on playgrounds, they were less likely to be playing on the playground’s floor.

With respect to mouthing activity, only one child touched the playground floor with their mouth, while four children touched the grass with their mouth while playing on turf. Age was an important factor for mouthing events, as age was negatively correlated with mouthing of hands, non-dietary objects, and all objects, regardless of the location where children played. This suggests that mouthing behavior decreases with age, which is supported by previously reported data. Although a similar grouped analysis conducted by Beamer et al. (2012) with the same data set in outdoor environments did not find significant differences of mouthing activity between younger (under 7 years of age) and older (7 years of age or 7–12 years) children, we observed significant differences in the mouthing behavior between younger and older children when they played on turf or playground structures [13]. Younger children (under 7 years old) playing on turf had significantly longer mouthing contact durations with non-dietary objects and all objects than older children. This suggests that longer mouthing durations by younger children may result in higher amounts of contaminant being ingested, as a longer exposure duration could increase the non-dietary ingestion of the chemical residue present in the hands/objects. Although children above 10 years of age who played on turf-like surfaces had a significantly higher mouthing duration with hands and non-dietary objects compared to some younger children (2–10 years old), we cannot explain this difference, but it is important to mention that this observation was recorded from a small group of six children (11–16 years old), so more data need to be collected from this age group. In addition, even though fewer children over the age of seven played on playgrounds, we found that when older children played on playgrounds, they had significantly fewer mouthing events and shorter mouthing durations with hands, non-dietary objects, and all objects than younger children (under 7 years old). Hence, more data are needed to evaluate the effect of age on children’s durations and to confirm statistically significant differences between children age groups.

Finally, our study did not find significant hand contact activity differences between female and male children regardless of the location where they played. Although there were no significant gender differences of the hand activity data and most mouthing activity data, girls had significantly longer mouthing contact durations with all objects while playing only on playgrounds but not on turf. This may indicate that future models should take into consideration not only the frequency but the mouthing duration in relation to gender and age when evaluating exposure to artificial turf. The analysis of the MLATS data of children’s mouthing and hand-to-object and -surface contacts on a specific activity basis (playing on playgrounds or turf), provides crucial information that can be used for the development of accurate assessment models that focus on exposure to artificial turf.

The quantified MLATS data collected in this study can be used to determine exposures that could negatively affect children’s health from playing on surfaces made with rubber crumb. Our average estimated daily incidental ingestion rate (0.08 g/day or 80 mg/day) among children playing on artificial turf is consistent with Peterson et al.’s (2018) rubber crumb ingestion rate (100 mg/day) for children spectating soccer games [2,25,35]. The estimated incidental ingestion rate calculated in this study would likely overestimate the consumption of crumb rubber among children, as some parameters were based on the assumption of soil adherence factors; however, soil particles are smaller than crumb rubber; consequently, ingestion rates for this material are likely lower than soil.

It is important to mention that the MLTAS data collected in this study can also be used to estimate other exposure routes such as dermal absorption. Hence, future studies should consider using these data to accurately determine children’s exposure to the chemicals and metals commonly present in recycled rubber crumb. Although synthetic turf has generally been used to replace natural grass on sport fields in North America and most exposure studies have focused on individuals playing sports, the application of synthetic turf as residential landscapes is increasing at a faster rate [36], which puts children conducting non-sport activities on these landscapes at risk of exposure to synthetic turf materials. Hence, the data presented in this study and the estimated incidental ingestion rate are important, as it allows us to better understand the exposure to artificial turf-like surfaces among children engaged in non-sporting playful activities.

One important limitation of this study was that the MLATS data were mainly not collected from videotapes of children playing on actual artificial turf or playgrounds with PIP rubber floors, but only two videotapes had children playing on rubber mulch nuggets. However, our study assumed that touching of floors and grass by children is a reasonable surrogate for touching of surfaces artificially made of recycled crumb rubber. Another limitation was that some of our assumptions were based on exposure to soil; for example, in the absence of crumb rubber adherence factors, we used a soil adherence factor because there are no available values for recycle crumb rubber commonly applied on artificial turf. Even though recycled crumb rubber particles are larger than soil particles and soil texture is different than crumb rubber [2], our results provide a framework to use our valuable micro-activity data of children playing (non-sporting activities) on turf-like surfaces for future risk assessment models associated with artificial turf.

An additional limitation is the small sample size, as our analysis corresponds to already recorded videotapes. Thus, there is a need to collect additional MLATS data of children playing on artificial turf and playgrounds with PIP rubber flooring or rubber mulch. An important study limitation is that all of our MLTAS data were collected only among doers (e.g., children playing on a playground area), and we were not able to compare the ingestion rate of rubber crumb with non-doers. In addition, we recognized that the event frequency could be different among seasons, as children’s playtime might increase on sunny days. Although future studies collecting MLTAS data should consider integrating a seasonality factor, our MLTAS data were not adjusted by seasons, as the data were collected during a regular academic year in the Bay Area of California, a region with fairly mild weather all year round in comparison to other areas of the country. Finally, another limitation was that we only analyzed data collected outdoors, as activity patterns often differ between indoor and outdoor environments. Therefore, future studies should consider collecting MLATS data in relation to artificial turf in indoor sport fields as well as children’s playmats made with recycled tires in other indoor environments.

## 5. Conclusions

The results of this study suggest that mouthing behavior decreases significantly with age and that environmental settings (i.e., playground or turf) can be important factors to consider in future studies. In addition, the mouthing duration with all objects was significantly longer for girls playing on playgrounds compared to boys. On the other hand, age and gender were not significantly associated with hand-to-object contact activity of children playing on turf or playgrounds. Moreover, the collected MLATS data allowed us to estimate the incidental ingestion rate of rubber crumb, which was consistent with previous studies. Besides reporting factors that may be associated with hand activity and mouthing behavior data, this study should help improve future risk assessments of artificial turf and playground mats by providing detailed summaries of MLATS activity data collected from children playing on playgrounds and turf. The data and results provided in this study can inform and assist regulatory agencies on the reduction of uncertainties when conducting chemical risk assessments.

## Figures and Tables

**Table 1:** Selected categories for objects/surfaces on turf and playgrounds and assumptions of estimated incidental ingestion.

***Super Categories***	**Virtual Timing Device Palette Categories**				
*Location*					
Turf	Yard, park, and garden				
*Sub-location*					
Playground	Playground structures				
*Object/Surface*					
Grass ^+^	Grass				
Dietary objects	Water/beverage, sticky food, other food, and food containers				
Non-dietary	Everything but dietary objects				
Hands *	Hands				
Floor ^ɫ^	Dirt, asphalt, rock, wood, tile, carpet/mat, sand, wood chips, rubber mulch, grass, and poured-in-placed (PIP) rubber				
All objects	Wood walls, wood tools, wood toys, vegetation, hard toys, porous plastic toys, fabric toys, plastic tools, plastic walls, paper, pool water, puddle water, metal walls, metal tools, footwear, carpet, wood floors, tile floors, rock floors, sidewalks, and dirt				
**Assumptions of Estimating Incidental Crumb Rubber Ingestion**					
Receptor by Age	Child 0 < 2 years	Child 2 < 6 years	Child 6 < 11 years	Units	Reference
AF_hand_	0.026			mg/cm^2^	Kissel et al., 1996 [22]
AF_obj_	0.026			mg/cm^2^	Kissel et al., 1996 [22]
TF_HTM_	0.5			unitless	OEHHA, 2008; OEHHA, 2011 [23,24]
TF_obj_	0.5			unitless	OEHHA, 2008; OEHHA, 2011 [23,24]
TF_indirect_	0.25			unitless	OEHHA, 2008; OEHHA, 2011 [23,24]
SA_HTM_, mean	26	34	53	cm^2^	OEHHA, 2008 [23]
SA_hands_, mean	256	379	525	cm^2^	USEPA, 2011 (Table ES-1, chapter 7) [25]
SA_head_, mean	806	653	681	cm^2^	USEPA, 2011 (Table ES-1, chapter 7) [25]
SA_obj_, mean	90	66	76	cm^2^	USEPA, 2011 (Table ES-1, chapter 7) [25]
AET, mean	408	474	438	h/year	USEPA, 2011 (Table ES-1, chapter 16) [25]
CF1	0.001			g/mg	
CF2	1/365			year/days	

^+^ Only analyzed when children played on natural grass or turf; * only analyzed for mouth contact files; ^ɫ^ only used when children played on playgrounds (Table S2). AET = annual event time; AF = adherence factor; SA = surface area; TF = transferred fraction; HTM = hand to mouth; obj = object.

**Table 2:** Children’s videotaped time spent on turf (n = 56) and playgrounds (n = 24) and the number of children.

	**Total Time on Turf**			**Time on Turf**		
	**Hand and/or Mouth in View**			**Hand and/or Mouth Not in View**		
Total turf time (min)	1812			99.5		
Median turf time (min) per child	84			2.5		
Percentage of time spent on turf (%)	71.1			3.9		
	**Total Time on Playgrounds** **Hand and/or Mouth in View**			**Time on Playgrounds** **Hand and/or Mouth Not in View**		
Total playground time (min)	531			38.2		
Median playground time (min) per child	21			0.3		
Percentage of time spent on playground (%)	20.8			1.5		
**Number of Children Playing on Turf and Playgrounds Grouped by Gender and US EPA Age Categories**						
	Age Groups (Turf)					
Age (years)	1 to <2	2 to <3	3 to <6	6 to <11	11 to <16	Total
Males	5	5	7	5	5	27
Females	3	2	10	13	1	29
Total	8	7	17	18	6	56
	Age Groups (Playgrounds)					
Age (years)	1 to <2	2 to <3	3 to <6	6 to <11	11 to <16	Total
Male	3	4	4	0	0	11
Female	2	1	4	6	0	13
Total	5	5	8	6	0	24

**Table 3:** Both hands and mouthing activity frequency, hourly duration, and median duration of children on turf (n = 56).

**Both Hands Contact Frequency (Event/h)**					**Mouthing Contact Frequency (Event/h)**				
	Grass	Dietary	Non-Dietary	All Objects	Grass	Hands	Dietary	Non-Dietary	All Objects
Mean	14.5	6.1	276.8	282.9	0.1	11.7	14.8	22.0	36.8
SD	35.5	10.3	126.7	125.3	0.4	14.2	28.4	29.9	44.7
Median	4.1	0.7	236.3	246.3	0.0	7.6	0.8	10.9	20.4
Range	0.0–251.8	0–40.3	107.7–765.8	109.7–768.3	0.0–2.5	0.0–80.1	0.0–159.1	0.0–185.1	0.0–205.1
**Both Hands Hourly Duration (min/h)**					**Mouthing Hourly Duration (min/h)**				
	Grass	Dietary	Non-Dietary	All Objects	Grass	Hands	Dietary	Non-Dietary	All Objects
Mean	0.9	1.8	36.5	38.3	0.0	0.4	1.8	1.4	3.2
SD	1.9	3.1	13.7	14.6	0.0	0.7	5.3	4.7	7.4
Median	0.2	0.1	33.3	34.8	0.0	0.2	0.1	0.3	0.7
Range	0.0–12.0	0.0–12.1	20.7–92.9	22.4–105.1	0.0–0.1	0.0–3.5	0.0–36.5	0.0–34.7	0.0–39.7
**Both Hands Median Contact Duration (s)**					**Mouthing Median Contact Duration (s)**				
	Grass (n = 42)	Dietary (n = 28)	Non-Dietary (n = 56)	All Objects (n = 56)	Grass (n = 4)	Hands (n = 49)	Dietary (n = 3)	Non-Dietary (n = 52)	All Objects (n = 53)
Mean	2.7	7.5	3.3	3.3	1.4	1.9	7.4	1.8	2.9
SD	2.1	5.3	0.9	0.9	0.8	1.9	22.1	1.9	8.1
Median	2.0	6.8	3.0	3.0	1.0	1.0	2.0	1.0	1.0
Range	0.5–13.0	1.0–23.0	1.0–5.0	1.0–5.0	1.0–2.5	0.5–12.0	1.0–123.0	1.0–12.0	1.0–60.0

SD, standard deviation; h, hour; s, seconds.

**Table 4:** Both hands and mouthing activity frequency, hourly duration, and median duration of children on playgrounds (n = 24).

**Both Hands Contact Frequency (Event/h)**					**Mouthing Contact Frequency (Event/h)**				
	Floors	Dietary	Non-Dietary	All Objects	Floors	Hands	Dietary	Non-Dietary	All Objects
Mean	29.6	1.6	292.7	294.3	-	19.6	43.1	28.2	71.3
SD	48.0	3.87	160.8	160.4	-	20.3	112.3	46.8	114.3
Median	12.1	0.0	261.4	262.3	-	9.8	0.0	10.2	20.4
Range	0.0–196.4	0.0–15.8	30.6–634.2	30.6–634.2	0.0–2.3	1.4–67.5	0.0–378.9	0.0–218.2	1.4–378.9
**Both Hands Hourly Duration (min/h)**					**Mouthing Hourly Duration (min/h)**				
	Floors	Dietary	Non-Dietary	All Objects	Floors	Hands	Dietary	Non–Dietary	All Objects
Mean	1.9	0.4	32.4	32.9	-	1.0	1.5	1.7	3.2
SD	2.9	1.3	4.4	4.6	-	1.6	4.1	4.1	5.5
Median	0.6	0.0	32.8	33.7	-	0.1	0.0	0.0	0.0
Range	0.0–10.1	0.0–5.0	22.9–39.7	22.9–39.7	0.0–0.1	0.0–5.0	0.0–16.5	0.0–20.0	0.0–20.0
**Both Hands Median Contact Duration (s)**					**Mouthing Median Contact Duration (s)**				
	Floors (n = 17)	Dietary (n = 6)	Non-Dietary (n = 24)	All Objects (n = 24)	Floors (n = 1)	Hands (n = 14)	Dietary (n = 6)	Non-Dietary (n = 13)	All Objects (n = 20)
Mean	3.3	11.8	3.9	3.9	-	2.1	25.6	2.2	7.8
SD	2.3	18.9	2.2	2.2	-	1.8	59.0	1.9	26.1
Median	2.0	2.3	4.0	4.0	-	1.0	1.5	1.0	1.0
Range	1.0–10.5	1.0–49.0	1.0–12.5	1.0–12.5	0.0–1.0	1.0–6.5	1.0–146.0	1.0–6.5	1.0–118.5

SD, standard deviation; h, hour; s, seconds.

**Table 5:** Estimated children’s indirect ingestion rates.

		Age 0–<2 years	Age 2–<6 years	Age 6–<11 years
*IR_HTM_* (g_crumb rubber_/h)	Mean	0.01	0.01	0.01
	Median	0.00	0.01	0.01
	95th Percentile	0.02	0.03	0.04
*IR_OTM_* (g_crumb rubber_/h)	Mean	0.04	0.03	0.03
	Median	0.02	0.01	0.02
	95th Percentile	0.13	0.09	0.11
*IR_HTOTM_* (g_crumb rubber_/h)	Mean	0.01	0.01	0.02
	Median	0.00	0.00	0.00
	95th Percentile	0.04	0.06	0.09
*IR_indirect_* (g_crumb rubber_/h)	Mean	0.06	0.05	0.06
	Median	0.03	0.02	0.03
	95th Percentile	0.19	0.18	0.24
*IR_daily_ing_* (g_crumb rubber_/day)	Mean	0.08	0.07	0.08
	Median	0.04	0.03	0.04
	95th Percentile	0.22	0.23	0.29

*IR_HTM_* = crumb rubber indirectly ingested via hand to mouth; *IR_OTM_* = crumb rubber indirectly ingested via the object to mouth; *IR_HTOTM_* = crumb rubber indirectly ingested via hand to object to mouth; *IR_indirect_* = total indirect ingestion rate; *IR_daily_ing_* = daily indirect ingestion rate.

## Data Availability

Summary of the data can be found in the Supplementary Materials.

## Supplementary Materials

The following supporting information can be downloaded at: https://www.mdpi.com/article/10.3390/ijerph19042483/s1, Table S1. Right hand contact frequency (event/h) while playing on the turf (n = 56); Table S2. Left hand contact frequency (event/h) while playing on the turf (n = 56); Table S3. Both hands object/surface contact frequency (event/h) (n = 56); Table S4. Right hand hourly contact duration (min/h) while playing on the turf (n = 56); Table S5. Left hand hourly contact duration (min/h) while playing on the turf (n = 56); Table S6. Hourly object/surface contact duration for both hands while playing on turf (min/h) (n = 56); Table S7. Right hand median contact duration while playing on the turf (s); Table S8. Left hand median contact duration while playing on the turf (s); Table S9. Both hands median contact duration while playing on the turf (s); Table S10. Spearman rank correlation for age (years) and both hands contact events; Table S11. Statistical analysis of hand contact events by EPA age groups. P-value from Kruskal-Wallis Test; Table S12. Statistical analysis of hand contact events younger (1–6 years) and older (7–12 years), P-value from Kruskal-Wallis Test; Table S12-A. Both hands contact frequency (event/h) while playing on turf by younger (1–6 years) and older (7–12 years); Table S12-B. Both hands hourly contact duration (min/h) while playing on turf by younger (1–6 years) and older (7–12 years); Table S12-C. Both hands median contact duration (s) while playing on turf by younger (1–6 years) and older (7–12 years); Table S13. Both hands contact frequency(event/h) while playing on turf by gender; Table S14. Both hands hourly contact duration (min/h) while playing on turf by gender; Table S15. Both hands contact median duration (s) while playing on turf by gender; Table S16. Spearman rank correlation for age (1–12 years) and mouthing events while playing on turf; Table S17. Mouthing contact frequency (event/h) while playing on turf by EPA age groups; Table S18. Mouthing hourly contact duration (min/h) while playing on turf by EPA age groups; Table S19. Mouthing contact median duration (s) while playing on turf by EPA age groups; Table S20. Mouthing contact median duration (s) while playing on turf by younger (1–6 years and older (7–12) children; Table S21. Mouthing contact frequency (event/h) while playing on turf by gender; Table S22. Mouthing contact duration (min/h) while playing on turf by gender; Table S23. Mouthing median duration (s) by gender; Table S24. Right hand frequency (event/h) while playing on the playground (n = 24); Table S25. Left hand frequency while playing on playground (n = 24); Table S26. Both hands object/surface contact frequency (event/h) on playground (n = 24); Table S27. Hourly object/surface contact duration (min/h) for right hand while playing on play-ground (n = 24); Table S28. Hourly object/surface contact duration (min/h) for left hand while playing on playground (n = 24); Table S29. Hourly object/surface contact duration (min/h) for both hands while playing on playground (n = 24); Table S30. Right hand median contact duration (s) while playing on the Playground; Table S31. Left hand median contact duration (s) while playing on the Playground; Table S32. Both hands object/surface median duration (s) while playing on the Playground; Table S33. Spearman rank correlation for both hand events and age (years) while playing on playgrounds; Table S33-A. Both hands contact frequency (event/h) on playground (n = 24) while playing on playground by younger (1–6 years) and older (7–12 years); Table S33-B. Both hands hourly contact duration (min/h) on playground (n = 24) while playing on playground by younger (1–6 years) and older (7–12 years); Table S33-C. Both hands contact median duration (s) on playground (n = 24) while playing on playground by younger (1–6 years) and older (7–12 years); Table S34. Both hands contact frequency while children play on playgrounds by gender (n = 24); Table S35. Both hands hourly contact duration (min/h) while children play on playgrounds by gender (n = 24); Table S36. Both hands median contact duration (s) while children play on playgrounds by gender; Table S37. Spearman rank correlation for age (years) and mouthing events while playing on playgrounds; Table S38. Mouthing contact frequency (event/h) while on playground (n = 24) by EPA age groups; Table S39. Mouthing hourly duration (min/h) while on playground (n = 24) by EPA age groups; Table S40. Mouthing median duration (s) while on playground by EPA age groups; Table S41. Mouthing contact frequency (event/h) while on playground (n = 24) by younger (1–6 years) and older (7–12 years); Table S42. Mouthing contact duration (min/h) while on playground (n = 24) by younger (1–6 years) and older (7–12 years); Table S43. Mouthing duration (min/h) while children play on playgrounds by gender; Table S44. Mouthing median duration (s) while children play on playgrounds by gender; Table S45. Mouthing frequency (event/h) while children play on playgrounds by gender; Figure S1. Median mouthing contact duration (s) of younger and older children while playing on turf.
