# Supplementary material for: Quantification and Analysis of Micro-Level Activities Data from Children Aged 1–12 Years Old for Use in the Assessments of Exposure to Recycled Tire on Turf and Playgrounds
Source: Int J Environ Res Public Health. 2022 Feb 21;19(4):2483. doi: 10.3390/ijerph19042483 (PMC8879270; doi:10.3390/ijerph19042483)
Supplement: Supplementary file 1 [file ijerph-19-02483-s001.zip › ijerph-1590436-supplementary.pdf]

International Journal of
Environmental Research and Public Health

MDPI

Article

Quantification and Analysis of Micro-Level Activities Data from Children Aged 1–12 Years Old for Use in the Assessments of Exposure to Recycled Tire on Turf and Playgrounds

Nicolas Lopez-Galvez [1,2,*], Jocelyn Claude [3], Patty Wong [3], Asa Bradman [4,5], Carly Hyland [4], Rosemary Castorina [4], Robert A. Canales [6], Dean Billheimer [1,7], Elmira Torabzadeh [7], James O. Leckie [8] and Paloma I. Beamer [1]

1 Mel and Enid Zuckerman College of Public Health, University of Arizona, Tucson, AZ 85724, USA; dean.billheimer@arizona.edu (D.B.); pbeamer@arizona.edu (P.I.B.)
2 San Diego State University Research Foundation, School of Public Health, San Diego, CA 92182, USA
3 Office of Environmental Health Hazard Assessment (OEHHA), California Environmental Protection Agency (EPA), Sacramento, CA 95812, USA; jocelyn.claude@oehha.ca.gov (J.C.); patty.wong@oehha.ca.gov (P.W.)
4 Center for Environmental Research and Children's Health, University of California, Berkeley, CA 94704, USA; abradman@berkeley.edu (A.B.); carlybarker@berkeley.edu (C.H.); rcastori@berkeley.edu (R.C.)
5 Department of Public Health, School of Social Sciences, Humanities, and Arts, University of California, Merced, CA 95343, USA
6 Department of Environmental and Occupational Health, Milken Institute School of Public Health, George Washington University, Washington, DC 20052, USA; rcanales@email.gwu.edu
7 Center for Biomedical Informatics and Biostatistics, University of Arizona, Tucson, AZ 85724, USA; etorabzadeh@gmail.com
8 Department of Civil and Environmental Engineering, Stanford University, Stanford, CA 94305, USA; leckie@stanford.edu
* Correspondence: Correspondence: nilopez@sdsu.edu or lopezgalvez@email.arizona.edu

Citation: Lopez-Galvez, N.; Claude, J.; Wong, P.; Bradman, A.; Hyland, C.; Castorina, R.; Canales, R.A.; Billheimer, D.; Torabzadeh, E.; Leckie, J.O.; et al. Quantification and Analysis of Micro-Level Activities Data from Children Aged 1–12 Years Old for Use in the Assessments of Exposure to Recycled Tire on Turf and Playgrounds. *Int. J. Environ. Res. Public Health* **2022**, *19*, 2483. https://doi.org/10.3390/ijerph19042483

Academic Editor: Paul B. Tchounwou

Received: 25 January 2022
Accepted: 18 February 2022
Published: 21 February 2022

Publisher's Note: MDPI stays neutral with regard to jurisdictional claims in published maps and institutional affiliations.

Copyright: © 2022 by the authors. Licensee MDPI, Basel, Switzerland. This article is an open access article distributed under the terms and conditions of the Creative Commons Attribution (CC BY) license (https://creativecommons.org/licenses/by/4.0/).

Supplementary Materials

The current supplementary information provides an additional summary of the analyzed micro-activity data of children playing on artificial turf like and playground structures. There is information on right/left hand along with both hands contact activity data. Data on mouthing events, duration and median duration is summarize as well. Results of spearman correlations and Kruskal Wallis test are also presented here. Finally, the results of the MLTAS data analyzed by several age groups and gender are presented in this summary.

Turf:

Table S1. Right hand contact frequency (event/h) while playing on the turf (n = 56).

	Grass	Dietary	Non-Dietary	All objects
Mean	7.20	3.10	129.40	132.50
SD	17.50	5.40	54.60	54.30
Min	0.00	0.00	57.30	57.30
p25	1.51	0.00	93.92	96.00
Median	3.73	0.14	112.50	115.68
p75	10.00	3.28	151.30	155.68
p95	26.50	15.34	221.12	221.12
p99	89.65	20.68	297.78	298.40
Max	122.33	20.72	371.99	373.36

Table S2. Left hand contact frequency (event/h) while playing on the turf (n = 56).

	Grass	Dietary	Non-Dietary	All objects
Mean	7.00	3.10	134.20	137.30
SD	18.20	5.20	68.10	67.40
Min	0.00	0.00	9.60	9.60
p25	1.22	0.00	101.04	103.84
Median	3.55	0.15	118.70	127.17
p75	8.03	4.80	145.58	149.58
p95	30.91	14.92	242.44	242.72
p99	90.08	19.62	376.70	377.20
Max	129.54	19.64	393.85	394.96

Table S3. Both hands object/surface contact frequency (event/h) (n = 56).

	Grass	Dietary	Non-Dietary	All objects
Mean	14.50	6.10	276.80	282.90
SD	35.50	10.30	126.70	125.30
Min	0.00	0.00	107.70	109.70
p25	0.78	0.00	192.78	205.57
Median	4.14	0.71	236.31	246.32
p75	14.93	8.82	308.21	324.38
p95	48.57	27.03	503.25	504.34
p99	151.84	40.30	718.79	719.90
Max	251.87	40.33	765.84	768.32
p-value *	0.7249	0.935	0.535	0.546

* from Wilcoxon Signed Rank test comparison of left- and right-hand contact frequency.

Table S4. Right hand hourly contact duration (min/h) while playing on the turf (n = 56).

	Grass	Dietary	Non-Dietary	All Objects
Mean	0.44	1.75	33.19	34.94
SD	0.97	3.08	9.61	9.04
Min	0.00	0.00	11.88	11.88
p25	0.00	0.00	26.33	29.04
Median	0.09	0.01	32.21	34.22
p75	0.36	2.41	39.14	40.28
p95	2.40	8.62	48.93	50.22
p99	5.87	11.99	53.06	55.06

Max	5.87	11.99	55.06	55.06

Table S5. Left hand hourly contact duration (min/h) while playing on the turf (n = 56).

	Grass	Dietary	Non-Dietary	All objects
Mean	0.45	1.61	31.94	33.55
SD	0.97	2.77	10.19	9.90
Min	0.00	0.00	5.68	5.68
p25	0.01	0.00	26.48	27.20
Median	0.11	0.01	31.23	32.94
p75	0.37	2.07	38.01	41.04
p95	2.20	7.46	48.49	48.49
p99	6.09	9.93	57.64	57.64
Max	6.09	9.93	57.64	57.64

Table S6. Hourly object/surface contact duration for both hands while playing on turf (min/h) (n = 56).

	Grass	Dietary	Non-Dietary	All objects
Mean	0.93	1.81	36.57	38.38
SD	1.97	3.08	13.71	14.60
Min	0.00	0.00	20.65	22.36
p25	0.05	0.00	29.26	31.55
Median	0.24	0.12	33.33	34.83
p75	1.22	2.47	35.98	36.97
p95	4.63	8.10	64.64	67.61
p99	8.02	11.90	81.66	87.12
Max	11.96	12.14	92.96	105.10
p-value *	0.876	0.265	0.167	0.071

* from Wilcoxon Signed Rank test comparison of left- and right-hand contact frequency.

Table S7. Right hand median contact duration while playing on the turf (s).

	Grass	Dietary	Non-Dietary	All Objects
n *	38	28	56	56
Mean	2.54	8.07	3.37	3.44
SD	1.48	6.11	1.08	1.10
Min	0.50	1.00	1.00	1.00
p25	1.50	4.00	3.00	3.00
Median	2.00	6.50	3.00	3.00
p75	3.00	11.63	4.00	4.00
p95	5.00	16.65	5.00	5.00
p99	6.83	24.30	5.73	5.73
Max	8.00	27.00	6.00	6.00

*Only participants who contacted object included in calculation of median contact duration.

Table S8. Left hand median contact duration while playing on the turf (s).

	Grass	Dietary	Non-Dietary	All objects
n *	42	28	56	56
Mean	3.52	7.57	3.28	3.29
SD	4.19	5.63	0.89	0.87
Min	0.50	1.00	2.00	2.00
p25	2.00	3.00	3.00	3.00

Median	2.00	6.50	3.00	3.00
p75	3.00	10.38	4.00	4.00
p95	12.33	18.13	5.00	4.63
p99	19.48	20.46	5.23	5.23
Max	20.50	21.00	5.50	5.50

* Only participants who contacted object included in calculation of median contact duration.

Table S9. Both hands median contact duration while playing on the turf (s).

	Grass	Dietary	Non - Dietary	All objects
n *	42	28	56	56
Mean	2.72	7.47	3.28	3.31
SD	2.05	5.25	0.92	0.95
Min	0.50	1.00	1.00	1.00
p25	2.00	4.00	3.00	3.00
Median	2.00	6.75	3.00	3.00
p75	3.00	10.00	4.00	4.00
p95	5.00	18.00	5.00	5.00
p99	13.00	23.00	5.00	5.00
Max	13.00	23.00	5.00	5.00
p-value +	0.815	0.367	0.611	0.245

*Only participants who contacted object included in calculation of median contact duration.
+Wilcoxon Signed Rank test comparison of left- and right-hand contact frequency.

Table S10. Spearman rank correlation for age (years) and both hands contact events.

Activity Variables	r	p-value
Hand contact frequency (event/h) (n = 56)		
Grass	0.1371	0.314
Non-Dietary	0.0839	0.538
Dietary	−0.003	0.983
All objects	0.087	0.523
Hourly hand contact duration (min/h) (n = 56)		
Grass	0.113	0.406
Non-Dietary	0.098	0.471
Dietary	−0.083	0.542
All objects	0.139	0.306
Hand contact median duration (s)		
Grass (n = 42)	0.137	0.314
Non-Dietary (n = 56)	0.084	0.538
Dietary (n = 28)	−0.003	0.983
All objects (n = 56)	0.087	0.523

Table S11. Statistical analysis of hand contact events by EPA age groups. p-value from Kruskal-Wallis Test.

Activity Variables	p-value
Hand contact frequency (event/h) (n = 56)	
Grass	0.361
Non-Dietary	0.397
Dietary	0.644

All objects	0.058
Hourly hand contact duration (min/h) (n = 56)	
Grass	0.252
Non-Dietary	0.132
Dietary	0.644
All objects	0.165
Median hand contact duration (s)	
Grass (n = 42)	0.286
Non-Dietary (n=56)	0.116
Dietary (n = 28)	0.491
All objects (n=56)	0.165

Table S12. Statistical analysis of hand contact events younger (1–6 years) and older (7–12 years). p-value from Kruskal-Wallis Test.

Activity Variables	p-value
Hand contact frequency (event/h) (n = 56)	
Grass	0.318
Non-Dietary	0.581
Dietary	0.585
All objects	0.584
Hourly hand contact duration (min/h) (n = 56)	
Grass	0.295
Non-Dietary	0.264
Dietary	0.328
All objects	0.191
Median hand contact duration (s)	
Grass (n = 42)	0.884
Non-Dietary (n = 56)	0.061
Dietary (n = 28)	0.092
All objects (n = 56)	0.069

Table S12-A. Both hands contact frequency (event/h) while playing on turf by younger (1–6 years) and older (7–12 years).

Age Group		Grass	Dietary	Non - Dietary	All Objects
1–6 years n = 32	Mean	9.2	6.4	280.8	287.2
	SD	14.5	10.6	147.8	145.6
	Min	0.0	0.0	107.7	109.7
	p25	0.6	0.0	192.1	203.1
	Median	3.2	0.9	223.3	228.4
	p75	14.0	8.8	307.9	308.4
	p95	37.2	27.9	680.3	680.3
	p99	70.0	40.3	765.8	768.3
	Max	70.0	40.3	765.8	768.3
7–12 years n = 24	Mean	21.6	5.7	271.4	277.1
	SD	51.3	10.2	94.4	94.5
	Min	0.0	0.0	138.8	138.8

p25	1.1	0.0	202.4	213.9
Median	6.9	0.3	252.5	252.8
p75	15.8	8.6	314.9	345.1
p95	59.2	26.5	473.6	473.6
p99	251.9	40.3	494.9	494.9
Max	251.9	40.3	494.9	494.9

Table S12-B. Both hands hourly contact duration (min/h) while playing on turf by younger (1–6 years) and older (7–12 years).

Age Group		Grass	Dietary	Non - Dietary	All Objects
1–6 years n = 32	Mean	0.8	2.1	30.8	36.5
	SD	1.5	3.3	10.9	11.8
	Min	0.0	0.0	15.6	22.4
	p25	0.0	0.0	23.7	29.6
	Median	0.2	0.1	28.6	34.5
	p75	0.6	3.9	33.2	36.4
	p95	4.6	8.2	59.7	68.7
	p99	4.8	11.7	62.2	72.4
	Max	4.8	11.7	62.2	72.4
7–12 years n = 24	Mean	1.2	1.4	35.4	40.8
	SD	2.5	2.7	18.7	17.7
	Min	0.0	0.0	14.7	22.9
	p25	0.1	0.0	21.0	32.3
	Median	0.3	0.0	33.0	35.6
	p75	0.9	2.2	35.7	37.2
	p95	4.2	5.5	65.3	67.3
	p99	12.0	12.1	96.0	105.1
	Max	12.0	12.1	96.0	105.1

Tables S12-C. Both hands median contact duration (s) while playing on turf by younger (1–6 years) and older (7–12 years).

Age Group		Grass	Dietary	Non - Dietary	All objects
1–6 years	n *	24	20	32	32
	Mean	2.65	8.45	3.44	3.47
	SD	1.59	5.69	0.80	0.80
	Min	0.50	1.00	2.00	2.00
	p25	2.00	5.00	3.00	3.00
	Median	2.00	7.25	3.50	4.00
	p75	3.00	10.00	4.00	4.00
	p95	5.00	20.50	5.00	5.00
	p99	8.00	23.00	5.00	5.00
	Max	8.00	23.00	5.00	5.00
7–12 years	n *	19	12	24	24
	Mean	2.82	5.83	3.06	3.10
	SD	2.57	4.12	1.05	1.10
	Min	1.00	1.00	1.00	1.00
	p25	2.00	2.75	2.00	2.00
	Median	2.00	4.50	3.00	3.00
	p75	3.00	8.25	4.00	4.00
	p95	13.00	14.00	5.00	5.00

	p99	13.00	14.00	5.00	5.00
	Max	13.00	14.00	5.00	5.00

*Only participants who contacted object included in calculation of median contact duration.

Table S13. Both hands contact frequency(event/h) while playing on turf by gender.

Gender		Grass	Dietary	Non - Dietary	All objects
Male n = 27	Mean	12.54	5.11	267.70	272.80
	SD	15.15	9.45	137.05	137.48
	Min	0.00	0.00	107.69	109.66
	Median	1.08	0.00	189.18	190.04
	p25	7.34	0.86	227.39	228.25
	p75	17.43	4.36	300.83	320.07
	p95	45.03	26.61	528.37	532.73
	p99	59.17	40.33	765.84	768.32
	Max	59.17	40.33	765.84	768.32
Female n = 29	Mean	16.38	6.98	285.27	292.25
	SD	47.47	11.14	118.16	114.49
	Min	0.00	0.00	163.11	173.66
	Median	0.34	0.00	211.05	222.22
	p25	3.78	0.00	253.24	253.24
	p75	9.70	8.86	325.19	349.05
	p95	70.00	27.91	494.87	494.87
	p99	251.87	40.28	680.29	680.29
	Max	251.87	40.28	680.29	680.29
p-value *		0.242	0.621	0.321	0.216

*p-value from Wilcoxon sum rank

Tables S14. Both hands hourly contact duration (min/h) while playing on turf by gender.

Gender		Grass	Dietary	Non - Dietary	All objects
Male n = 27	Mean	0.94	1.86	33.78	39.52
	SD	1.40	3.52	17.40	18.16
	Min	0.00	0.00	14.72	22.36
	Median	0.09	0.00	23.44	30.02
	p25	0.40	0.13	29.74	34.01
	p75	0.96	2.36	34.97	37.26
	p95	4.63	11.71	62.19	72.41
	p99	4.79	12.14	96.03	105.10
	Max	4.79	12.14	96.03	105.10
Female n = 29	Mean	0.92	1.76	31.82	37.32
	SD	2.41	2.66	12.10	10.48
	Min	0.00	0.00	16.75	23.24
	Median	0.01	0.00	23.47	31.58
	p25	0.11	0.11	29.83	35.19
	p75	0.38	2.80	34.18	36.95
	p95	4.63	8.08	62.51	65.20
	p99	11.96	8.16	65.28	67.25
	Max	11.96	8.16	65.28	67.25
p-value		0.059	0.679	0.974	0.486

*p-value from Wilcoxon sum rank

Table S15. Both hands contact median duration (s) while playing on turf by gender.

Gender		Grass	Dietary	Non - Dietary	All objects
Male	n	22	27	27	27
	Mean	2.70	16.67	62.50	79.17
	SD	1.67	45.85	30.09	52.22
	Min	0.50	0.00	22.75	34.00
	Median	2.00	0.00	39.00	50.50
	p25	2.00	1.50	57.00	59.00
	p75	3.00	19.00	80.00	87.00
	p95	5.00	31.50	114.50	173.00
	p99	8.00	241.00	159.00	282.50
	Max	8.00	241.00	159.00	282.50
Female	n	21	29	29	29
	Mean	2.54	9.69	77.80	87.49
	SD	2.12	33.31	54.47	65.71
	Min	0.50	0.00	22.50	22.50
	Median	1.50	0.00	52.25	52.25
	p25	2.00	0.00	61.00	74.50
	p75	2.50	16.00	85.25	90.00
	p95	4.00	42.50	135.50	191.50
	p99	13.00	56.00	415.50	445.00
	Max	13.00	56.00	415.50	445.00
p-value *		0.324	0.567	0.358	0.566

*p-value from Wilcoxon sum rank.

Table S16. Spearman rank correlation for age (1–12 years) and mouthing events while playing on turf.

Activity Variables	r	p-value
Mouth contact frequency (event/h) (n = 56)		
Grass	−0.221	0.101
Hands	−0.033	0.811
Non-Dietary	−0.124	0.364
Dietary	−0.058	0.672
All objects	−0.160	0.241
Mouth contact duration (min/h) (n = 56)		
Grass	−0.221	0.102
Hands	−0.194	0.151
**Non-Dietary ***	**−0.308**	**0.021**
Dietary	−0.067	0.625
**All objects ***	**−0.266**	**0.048**
Mouth median duration (s)		
Grass (n = 4)	0.272	0.728
**Hands * (n = 49) ***	**−0.357**	**0.012**
**Non-Dietary * (n = 52)***	**−0.353**	**0.010**
Dietary (n = 30)	−0.072	0.707
All objects (n=53)	−0.254	0.067

* Significant ($p < 0.05$) correlation with age (Spearman's rank correlation).

Table S17. Mouthing contact frequency (event/h) while playing on turf by EPA age groups.

Age Group		Grass	Hands	Dietary	Non-Dietary	All Objects
< 2 (n = 8)	Mean	0.34	12.74	2.79	25.69	28.48
	SD	0.65	11.69	4.39	22.61	23.62
	Min	0.00	0.66	0.00	1.48	2.96
	Median	0.00	3.70	0.00	9.44	9.44
	p25	0.00	11.99	0.74	16.30	19.68
	p75	0.51	16.56	4.41	44.37	51.41
	p95	1.69	36.78	12.05	63.82	63.82
	p99	1.69	36.78	12.05	63.82	63.82
	Max	1.69	36.78	12.05	63.82	63.82
2 to < 3 (n = 7)	Mean	-	10.33	28.11	15.78	43.89
	SD	-	5.22	36.10	8.21	30.77
	Min	-	3.96	0.00	5.29	14.03
	Median	-	5.85	0.00	6.68	15.35
	p25	-	9.58	2.65	14.03	29.12
	p75	-	15.84	74.00	23.35	79.28
	p95	-	18.53	80.19	26.47	86.87
	p99	-	18.53	80.19	26.47	86.87
	Max	-	18.53	80.19	26.47	86.87
3 to < 6 (n = 17)	Mean	0.22	9.99	20.47	27.50	47.97
	SD	0.65	11.66	39.23	43.24	62.07
	Min	0.00	0.00	0.00	0.00	0.68
	Median	0.00	2.56	0.00	7.42	7.98
	p25	0.00	6.89	1.74	10.89	23.70
	p75	0.00	11.66	24.44	30.47	45.75
	p95	2.49	41.04	159.12	185.14	205.09
	p99	2.49	41.04	159.12	185.14	205.09
	Max	2.49	41.04	159.12	185.14	205.09
6 to < 11 (n =18)	Mean	-	6.74	8.26	12.21	20.46
	SD	-	5.48	15.73	17.48	25.25
	Min	-	0.00	0.00	0.00	0.00
	Median	-	0.54	0.00	5.86	5.86
	p25	-	7.12	0.00	7.79	8.87
	p75	-	9.00	7.76	10.85	28.12
	p95	-	19.25	48.83	76.19	76.19
	p99	-	19.25	48.83	76.19	76.19
	Max	-	19.25	48.83	76.19	76.19
11 to < 16 (n = 6)	Mean	-	31.22	19.15	38.08	57.24
	SD	-	29.72	27.42	34.06	58.80
	Min	-	1.23	0.00	2.47	5.01
	Median	-	4.01	1.00	4.01	11.73
	p25	-	27.12	9.02	38.14	42.53
	p75	-	47.70	23.44	57.40	80.84
	p95	-	80.11	72.43	88.34	160.77
	p99	-	80.11	72.43	88.34	160.77
	Max	-	80.11	72.43	88.34	160.77
p-value *		-	0.222	0.174	0.237	0.132

*p-value from Kruskal-Wallis Test.

Table S18. Mouthing hourly contact duration (min/h) while playing on turf by EPA age groups.

Age Group		Grass	Hands	Dietary	Non-Dietary	All Objects
< 2 (n = 8)	Mean	0.01	1.13	0.65	1.54	2.18
	SD	0.01	1.50	1.39	1.52	1.84
	Min	0.00	0.03	0.00	0.16	0.16
	Median	0.00	0.24	0.00	0.30	0.31
	p25	0.00	0.33	0.01	0.96	2.09
	p75	0.01	2.17	0.58	2.84	3.98
	p95	0.03	3.54	4.02	3.95	4.57
	p99	0.03	3.54	4.02	3.95	4.57
	Max	0.03	3.54	4.02	3.95	4.57
2 to < 3 (n = 7)	Mean	-	0.48	0.97	0.63	1.60
	SD	-	0.58	1.29	0.64	1.08
	Min	-	0.09	0.00	0.13	0.51
	Median	-	0.15	0.00	0.19	0.67
	p25	-	0.23	0.28	0.33	1.39
	p75	-	0.51	1.71	0.99	2.04
	p95	-	1.75	3.45	1.92	3.65
	p99	-	1.75	3.45	1.92	3.65
	Max	-	1.75	3.45	1.92	3.65
3 to < 6 (n = 17)	Mean	0.01	0.27	2.24	2.79	5.02
	SD	0.02	0.38	3.75	8.27	9.90
	Min	0.00	0.00	0.00	0.00	0.05
	Median	0.00	0.07	0.00	0.19	0.27
	p25	0.00	0.17	0.09	0.36	0.64
	p75	0.00	0.28	1.45	1.76	3.88
	p95	0.10	1.59	10.53	34.71	39.65
	p99	0.10	1.59	10.53	34.71	39.65
	Max	0.10	1.59	10.53	34.71	39.65
6 to < 11 (n =18)	Mean	-	0.16	2.42	0.40	2.82
	SD	-	0.14	8.58	0.90	8.57
	Min	-	0.00	0.00	0.00	0.00
	Median	-	0.04	0.00	0.04	0.04
	p25	-	0.12	0.00	0.13	0.20
	p75	-	0.23	0.23	0.27	0.88
	p95	-	0.52	36.52	3.92	36.66
	p99	-	0.52	36.52	3.92	36.66
	Max	-	0.52	36.52	3.92	36.66
11 to < 16 (n = 6)	Mean	-	0.96	1.21	1.14	2.35
	SD	-	0.89	1.89	1.08	2.63
	Min	-	0.02	0.00	0.05	0.30
	Median	-	0.15	0.15	0.15	0.60
	p25	-	0.79	0.54	0.92	1.18
	p75	-	1.75	1.06	2.07	3.77
	p95	-	2.25	4.99	2.71	7.06
	p99	-	2.25	4.99	2.71	7.06
	Max	-	2.25	4.99	2.71	7.06
p-value *		-	**0.025**	0.310	**0.016**	0.084

Table S19. Mouthing contact median duration (s) while playing on turf by EPA age groups.

Age Group		Grass	Hands	Dietary	Non-Dietary	All Objects
< 2	n	2	8	4	8	8
	Mean	1.00	3.69	6.88	3.44	2.63
	SD	0.00	3.83	5.81	3.90	2.15
	Min	1.00	1.00	1.00	1.00	1.00
	Median	1.00	1.00	2.25	1.00	1.00
	p25	1.00	2.50	6.25	2.00	2.00
	p75	1.00	4.75	11.50	4.25	3.75
	p95	1.00	12.00	14.00	12.00	6.50
	p99	1.00	12.00	14.00	12.00	6.50
	Max	1.00	12.00	14.00	12.00	6.50
2 to < 3	n	0	7	5	7	7
	Mean	-	1.71	2.20	1.64	1.71
	SD	-	0.91	1.10	0.75	0.76
	Min	-	1.00	1.00	1.00	1.00
	Median	-	1.00	2.00	1.00	1.00
	p25	-	1.50	2.00	1.50	2.00
	p75	-	2.00	2.00	2.00	2.00
	p95	-	3.50	4.00	3.00	3.00
	p99	-	3.50	4.00	3.00	3.00
	Max	-	3.50	4.00	3.00	3.00
3 to < 6	n	2	14	10	16	17
	Mean	1.75	1.61	3.40	1.94	1.85
	SD	1.06	1.13	2.49	1.25	1.09
	Min	1.00	0.50	1.00	0.50	0.50
	Median	1.00	1.00	2.00	1.00	1.00
	p25	1.75	1.00	2.75	1.75	2.00
	p75	2.50	2.00	4.00	2.50	2.00
	p95	2.50	5.00	8.50	5.00	4.00
	p99	2.50	5.00	8.50	5.00	4.00
	Max	2.50	5.00	8.50	5.00	4.00
6 to < 11	n	0	14	6	15	15
	Mean	-	1.29	21.92	1.27	5.27
	SD	-	1.07	49.53	1.10	15.18
	Min	-	1.00	1.00	0.00	0.00
	Median	-	1.00	1.00	1.00	1.00
	p25	-	1.00	1.75	1.00	1.00
	p75	-	1.00	3.00	1.00	2.00
	p95	-	5.00	123.00	5.00	60.00
	p99	-	5.00	123.00	5.00	60.00
	Max	-	5.00	123.00	5.00	60.00
11 to < 16	n	0	6	5	6	6
	Mean	-	1.50	3.60	1.42	1.58
	SD	-	0.55	3.05	0.49	0.49
	Min	-	1.00	2.00	1.00	1.00
	Median	-	1.00	2.00	1.00	1.00
	p25	-	1.50	2.00	1.25	1.75
	p75	-	2.00	3.00	2.00	2.00

	p95	-	2.00	9.00	2.00	2.00
	p99	-	2.00	9.00	2.00	2.00
	Max	-	2.00	9.00	2.00	2.00
p-value *		-	0.079	0.604	0.110	0.521

* p-value from Kruskal-Wallis Test (Only participants who contacted object included in calculation).

Table S20. Mouthing contact median duration (s) while playing on turf by younger (1–6 years and older (7–12) children.

Age Group		Grass	Hands	Dietary	Non-Dietary	All Objects
1–6 years	n *	4	34	22	36	37
	Mean	1.38	2.15	9.07	2.19	3.61
	SD	0.75	2.21	25.65	2.16	9.63
	Min	1.00	0.50	1.00	0.50	0.50
	p25	1.00	1.00	2.00	1.00	1.00
	Median	1.00	1.00	2.75	1.50	2.00
	p75	1.75	2.00	4.00	2.00	2.00
	p95	2.50	6.50	14.00	6.50	6.50
	p99	2.50	12.00	123.00	12.00	60.00
	Max	2.50	12.00	123.00	12.00	60.00
7–12 years	n *	0	15	8	16	16
	Mean	-	1.20	2.81	1.16	1.22
	SD	-	0.41	2.56	0.51	0.55
	Min	-	1.00	1.00	0.00	0.00
	p25	-	1.00	1.75	1.00	1.00
	Median	-	1.00	2.00	1.00	1.00
	p75	-	1.00	2.50	1.25	1.75
	p95	-	2.00	9.00	2.00	2.00
	p99	-	2.00	9.00	2.00	2.00
	Max	-	2.00	9.00	2.00	2.00
p-value *		-	0.079	0.377	**0.030**	**0.026**

* Wilcoxon sum rank test. Only participants who contacted object included in calculation of median contact duration.

Table S21. Mouthing contact frequency (event/h) while playing on turf by gender.

Gender		Grass	Hands	Dietary	Non-Dietary	All Objects
Males n = 27	Mean	0.10	12.76	14.30	19.97	34.28
	SD	0.37	16.79	22.93	22.12	37.66
	Min	0.00	0.00	0.00	0.00	0.00
	p25	0.00	3.25	0.00	4.01	6.35
	Median	0.00	8.14	1.48	13.31	22.77
	p75	0.00	16.61	30.91	26.47	59.22
	p95	1.02	41.04	72.43	63.82	106.17
	p99	1.69	80.11	74.00	88.34	160.77
	Max	1.69	80.11	74.00	88.34	160.77
Females n = 29	Mean	0.13	10.63	15.33	23.88	39.20
	SD	0.50	11.38	33.10	35.92	50.87
	Min	0.00	0.00	0.00	0.00	0.00
	p25	0.00	4.51	0.00	7.14	7.42
	Median	0.00	7.14	0.00	10.85	18.28

	p75	0.00	12.63	19.69	26.24	53.12
	p95	1.17	36.78	80.19	76.19	189.59
	p99	2.49	47.70	159.12	185.14	205.09
	Max	2.49	47.70	159.12	185.14	205.09
p-value *		0.971	0.825	0.412	0.737	0.941

*p-value from Wilcoxon sum rank.

Table S22. Mouthing contact duration (min/h) while playing on turf by gender.

Gender		Grass	Hands	Dietary	Non-Dietary	All Objects
Males n = 27	Mean	0.00	0.47	1.00	0.70	1.70
	SD	0.01	0.75	2.02	0.87	2.44
	Min	0.00	0.00	0.00	0.00	0.00
	p25	0.00	0.10	0.00	0.14	0.27
	Median	0.00	0.20	0.08	0.30	0.81
	p75	0.00	0.51	1.26	0.99	2.04
	p95	0.02	1.75	4.99	2.14	7.06
	p99	0.03	3.53	8.94	3.53	10.91
	Max	0.03	3.53	8.94	3.53	10.91
Females n = 29	Mean	0.00	0.45	2.55	2.03	4.59
	SD	0.02	0.78	7.08	6.39	9.82
	Min	0.00	0.00	0.00	0.00	0.00
	p25	0.00	0.10	0.00	0.12	0.16
	Median	0.00	0.22	0.00	0.29	0.50
	p75	0.00	0.30	1.06	1.67	3.88
	p95	0.02	2.25	10.53	3.95	36.66
	p99	0.10	3.54	36.52	34.71	39.65
	Max	0.10	3.54	36.52	34.71	39.65
p-value *		0.956	0.594	0.598	0.902	0.961

*p-value from Wilcoxon sum rank.

Table S23. Mouthing median duration (s) by gender.

Gender		Grass	Hands	Dietary	Non-Dietary	All Objects
Male	n *	2	25	17	25	26
	Mean	1.00	2.18	3.12	2.24	1.98
	SD	0.00	2.52	2.69	2.50	1.56
	Min	1.00	1.00	1.00	1.00	1.00
	p25	1.00	1.00	1.00	1.00	1.00
	Median	1.00	1.00	2.00	1.00	1.00
	p75	1.00	2.00	4.00	2.00	2.00
	p95	1.00	6.50	9.00	6.50	5.50
	p99	1.00	12.00	9.00	12.00	6.50
	Max	1.00	12.00	9.00	12.00	6.50
Female	n *	2	24	13	27	27
	Mean	1.75	1.52	13.00	1.54	3.76
	SD	1.06	0.81	33.24	0.91	11.27
	Min	1.00	0.50	1.00	0.00	0.00
	p25	1.00	1.00	2.00	1.00	1.00
	Median	1.75	1.00	3.00	1.00	2.00
	p75	2.50	2.00	3.50	2.00	2.00

p95	2.50	3.00	123.00	3.00	4.00
p99	2.50	3.50	123.00	4.00	60.00
Max	2.50	3.50	123.00	4.00	60.00
p-value *	0.317	0.718	0.241	0.512	0.962

*p-value from Wilcoxon sum rank.

Playground:

Table S24. Right hand frequency (event/h) while playing on the playground (n = 24).

	Floors	Dietary	Non-Dietary	All objects
Mean	15.69	0.56	148.11	148.68
SD	32.22	1.66	86.26	86.11
Min	0.00	0.00	15.88	15.88
p25	0.00	0.00	97.53	99.39
Median	4.81	0.00	128.13	128.13
p75	13.49	0.00	193.30	193.30
p95	69.23	3.71	327.27	327.27
p99	146.70	7.02	342.86	342.86
Max	146.70	7.02	342.86	342.86

Table S25. Left hand frequency while playing on playground (n = 24).

	Floors	Dietary	Non-Dietary	All objects
Mean	13.95	1.02	144.55	145.57
SD	20.58	2.83	79.16	78.79
Min	0.00	0.00	10.71	10.71
p25	0.00	0.00	95.60	95.60
Median	5.69	0.00	143.56	145.79
p75	15.55	0.00	174.49	175.07
p95	62.43	8.86	291.34	291.34
p99	72.00	10.77	337.96	337.96
Max	72.00	10.77	337.96	337.96

Table S26. Both hands object/surface contact frequency (event/h) on playground (n = 24).

	Floors	Dietary	Non-Dietary	All objects
Mean	29.64	1.58	292.67	294.25
SD	48.01	3.87	160.75	160.35
Min	0.00	0.00	30.60	30.60
p25	0.00	0.00	198.42	204.18
Median	12.12	0.00	261.38	262.28
p75	36.31	0.59	377.91	378.50
p95	141.23	10.77	602.49	602.49
p99	196.37	15.88	634.20	634.20
Max	196.37	15.88	634.20	634.20
p-value *	0.592	0.663	0.821	0.820

* from Wilcoxon Signed Rank test comparison of left- and right-hand contact.

Table S27. Hourly object/surface contact duration (min/h) for right hand while playing on playground (n = 24).

	Floors	Dietary	Non-Dietary	All Objects
Mean	1.04	0.25	16.50	16.74

SD	1.83	1.03	3.28	3.26
Min	0.00	0.00	3.64	3.64
p25	0.00	0.00	15.52	15.93
Median	0.23	0.00	17.21	17.52
p75	1.17	0.00	18.62	18.64
p95	6.13	0.75	19.23	19.23
p99	6.26	5.04	19.85	19.85
Max	6.26	5.04	19.85	19.85

Table S28. Hourly object/surface contact duration (min/h) for left hand while playing on playground (n = 24).

	Floors	Dietary	Non-Dietary	All Objects
Mean	0.85	0.19	16.05	16.23
SD	1.21	0.80	3.29	3.33
Min	0.00	0.00	4.38	4.38
p25	0.00	0.00	14.86	15.16
Median	0.19	0.00	16.97	17.15
p75	1.25	0.00	18.09	18.24
p95	3.54	0.43	19.82	19.82
p99	3.81	3.93	20.00	20.00
Max	3.81	3.93	20.00	20.00

Table S29. Hourly object/surface contact duration (min/h) for both hands while playing on playground (n = 24).

	Floors	Dietary	Non-Dietary	All Objects
Mean	1.96	0.43	32.44	32.87
SD	2.98	1.28	4.43	4.63
Min	0.00	0.00	22.98	22.98
p25	0.00	0.00	31.61	31.66
Median	0.58	0.00	32.76	33.66
p75	2.32	0.01	35.42	36.65
p95	9.67	3.93	38.59	38.59
p99	10.07	5.04	39.67	39.67
Max	10.07	5.04	39.67	39.67
p-value	0.753	0.737	0.275	0.216
* from Wilcoxon Signed Rank test comparison of left- and right-hand contact				

Table S30. Right hand median contact duration (s) while playing on the Playground.

	Floors	Dietary	Non-Dietary	All Objects
n	15	3	24	24
Mean	4.10	18.67	4.15	4.13
SD	2.35	26.31	2.31	2.31
Min	1.00	2.00	1.00	1.00
p25	2.50	3.50	3.00	3.00
Median	4.00	5.00	4.00	4.00
p75	5.25	27.00	5.00	5.00
p95	7.90	44.60	6.85	6.85
p99	9.58	48.12	11.24	11.24
Max	10.00	49.00	12.50	12.50

Table S31. Left hand median contact duration (s) while playing on the Playground.

	Floors	Dietary	Non-Dietary	All Objects
n	17	4	24	24
Mean	3.26	4.75	10.33	10.29
SD	2.89	6.18	23.87	23.89
Min	1.00	1.00	2.00	2.00
p25	1.50	1.75	3.00	3.00
Median	3.00	2.00	4.00	3.75
p75	4.00	5.00	5.00	5.00
p95	7.40	12.20	50.05	50.20
p99	11.88	13.64	97.66	97.66
Max	13.00	14.00	109.50	109.50

Table S32. Both hands object/surface median duration (s) while playing on the Playground.

	Floors	Dietary	Non-Dietary	All Objects
n	17	6	24	24
Mean	3.32	11.75	3.90	3.94
SD	2.32	18.89	2.24	2.25
Min	1.00	1.00	1.00	1.00
p25	2.00	2.00	3.00	3.00
Median	2.00	2.25	4.00	4.00
p75	4.00	11.13	4.00	4.13
p95	6.90	40.25	6.00	6.00
p99	9.78	47.25	11.01	11.01
Max	10.50	49.00	12.50	12.50
p-value	0.136	0.279	0.942	0.883

* from Wilcoxon Signed Rank test comparison of left- and right-hand contact.

Table S33. Spearman rank correlation for both hand events and age (years) while playing on playgrounds.

Activity Variables	r	p-value
Both hands contact frequency (event/h) (n = 24)		
Floors	−0.343	0.101
Non-Dietary	0.235	0.270
Dietary	0.054	0.801
All objects	−0.234	0.272
Both hands contact duration (min/h) (n = 24)		
Floors	−0.163	0.448
Non-Dietary	0.360	0.084
Dietary	0.061	0.776
All objects	0.391	0.059
Both hands median contact duration (s)		
Floors (n = 17)	0.274	0.287
Non-Dietary * (n = 24)	−0.072	0.737
Dietary (n = 7)	0.537	0.272
All objects * (n = 24)	−0.023	0.916

* Significant ($p < 0.05$) correlation with age (Spearman's rank correlation). .

Table S33-A. Both hands contact frequency (event/h) on playground (n = 24) while playing on playground by younger (1–6 years) and older (7–12 years).

Age Group		Floors	Dietary	Non-Dietary	All Objects
1–6 years (n = 21)	Mean	33.24	1.81	301.30	303.11
	SD	50.36	4.10	169.75	169.22
	Min	0.00	0.00	30.60	30.60
	p25	0.71	0.00	205.12	205.86
	Median	16.60	0.00	262.79	262.79
	p75	50.83	1.17	401.54	401.54
	p95	141.23	10.77	602.49	602.49
	p99	196.37	15.88	634.20	634.20
	Max	196.37	15.88	634.20	634.20
7–12 years (n = 3)	Mean	4.41	-	232.23	232.23
	SD	7.64	-	52.63	52.63
	Min	0.00	-	171.54	171.54
	p25	0.00	-	171.54	171.54
	Median	0.00	-	259.97	259.97
	p75	13.23	-	265.19	265.19
	p95	13.23	-	265.19	265.19
	p99	13.23	-	265.19	265.19
	Max	13.23	-	265.19	265.19
p-value		0.144	-	0.513	0.458

Table S33-B. Both hands hourly contact duration (min/h) on playground (n = 24) while playing on playground by younger (1–6 years) and older (7–12 years).

Age Group		Floors	Dietary	Non-Dietary	All Objects
1–6 years (n = 21)	Mean	2.19	0.49	31.98	32.48
	SD	3.12	1.36	4.42	4.70
	Min	0.00	0.00	22.98	22.98
	p25	0.01	0.00	31.56	31.56
	Median	0.78	0.00	32.20	33.51
	p75	2.88	0.02	34.39	36.34
	p95	9.67	3.93	37.10	37.24
	p99	10.07	5.04	39.67	39.67
	Max	10.07	5.04	39.67	39.67
7–12 years (n = 3)	Mean	0.30	-	35.67	35.67
	SD	0.51	-	3.47	3.47
	Min	0.00	-	31.83	31.83
	p25	0.00	-	31.83	31.83
	Median	0.00	-	36.59	36.59
	p75	0.89	-	38.59	38.59
	p95	0.89	-	38.59	38.59
	p99	0.89	-	38.59	38.59
	Max	0.89	-	38.59	38.59
p-value		0.178	-	0.206	0.316

Table S33-C. Both hands contact median duration (s) on playground (n = 24) while playing on playground by younger (1–6 years) and older (7–12 years).

Age Group		Floors	Dietary	Non-Dietary	All Objects
1–6 years	n*	16	3	21	21
	Mean	3.28	11.75	3.95	4.00
	SD	2.39	18.89	2.39	2.40
	Min	1.00	1.00	1.00	1.00
	p25	2.00	2.00	3.00	3.00
	Median	2.00	2.25	4.00	4.00
	p75	4.00	14.00	4.00	4.50
	p95	10.50	49.00	6.00	6.00
	p99	10.50	49.00	12.50	12.50
	Max	10.50	49.00	12.50	12.50
7–12 years	n*	2	0	3	3
	Mean	4.00	-	3.50	3.50
	SD	4.00	-	0.50	0.50
	Min	4.00	-	3.00	3.00
	p25	4.00	-	3.00	3.00
	Median	4.00	-	3.50	3.50
	p75	4.00	-	4.00	4.00
	p95	4.00	-	4.00	4.00
	p99	4.00	-	4.00	4.00
	Max	4.00	-	4.00	4.00
p-value		0.456	-	0.622	0.623

Table S34. Both hands contact frequency while children play on playgrounds by gender (n = 24).

Gender		Floors	Dietary	Non-Dietary	All objects
Male n = 11	Mean	42.16	0.36	272.99	273.35
	SD	66.00	0.88	180.55	180.58
	Min	0.00	0.00	30.60	30.60
	p25	0.00	0.00	80.59	80.59
	Median	16.60	0.00	275.85	275.85
	p75	58.06	0.00	401.54	401.54
	p95	196.37	2.80	602.49	602.49
	p99	196.37	2.80	602.49	602.49
	Max	196.37	2.80	602.49	602.49
Female n = 13	Mean	19.04	2.61	309.32	311.93
	SD	22.85	5.05	147.34	146.19
	Min	0.00	0.00	171.54	171.54
	p25	0.00	0.00	205.86	208.83
	Median	11.01	0.00	259.97	261.77
	p75	19.48	3.62	288.28	304.16
	p95	62.43	15.88	634.20	634.20
	p99	62.43	15.88	634.20	634.20
	Max	62.43	15.88	634.20	634.20
	p-value*	0.224	0.599	0.257	0.251

* from Wilcoxon Sum Rank test comparison of male and female.

Table S35. Both hands hourly contact duration (min/h) while children play on playgrounds by gender (n = 24).

Gender		Floors	Dietary	Non-Dietary	All objects
Male n = 11	Mean	2.51	0.01	32.71	32.72
	SD	3.31	0.03	4.43	4.43
	Min	0.00	0.00	22.98	22.98
	p25	0.00	0.00	31.56	31.56
	Median	0.78	0.00	32.20	32.20
	p75	5.60	0.00	36.34	36.34
	p95	9.67	0.09	39.67	39.67
	p99	9.67	0.09	39.67	39.67
	Max	9.67	0.09	39.67	39.67
Female n = 13	Mean	1.49	0.79	32.22	33.01
	SD	2.72	1.69	4.59	4.97
	Min	0.00	0.00	23.64	23.64
	Median	0.00	0.00	31.66	31.76
	p25	0.38	0.00	33.31	34.50
	p75	1.65	0.12	34.50	36.70
	p95	10.07	5.04	38.59	38.59
	p99	10.07	5.04	38.59	38.59
	Max	10.07	5.04	38.59	38.59
p-value *		0.630	0.224	0.599	0.257

* from Wilcoxon Sum Rank test comparison of male and female.

Table S36. Both hands median contact duration (s) while children play on playgrounds by gender.

Gender		Floors	Dietary	Non-Dietary	All objects
Males	n	8	2	11	11
	Mean	3.44	1.50	4.59	4.59
	SD	3.18	0.71	3.01	3.01
	Min	1.00	1.00	1.00	1.00
	Median	1.50	1.00	3.00	3.00
	p25	2.00	1.50	4.00	4.00
	p75	4.50	2.00	6.00	6.00
	p95	10.50	2.00	12.50	12.50
	p99	10.50	2.00	12.50	12.50
	Max	10.50	2.00	12.50	12.50
Females	n	9	4	13	13
	Mean	3.22	16.88	3.31	3.38
	SD	1.39	22.12	1.13	1.21
	Min	2.00	2.00	1.00	1.00
	Median	2.00	2.25	3.00	3.00
	p25	3.00	8.25	3.50	3.50
	p75	4.00	31.50	4.00	4.00
	p95	6.00	49.00	5.00	5.00
	p99	6.00	49.00	5.00	5.00
	Max	6.00	49.00	5.00	5.00
p-value *		0.753	0.638	0.675	0.573

* from Wilcoxon Sum Rank test comparison of male and female.

Table S37. Spearman rank correlation for age (years) and mouthing events while playing on playgrounds.

Activity Variables	r	p-value
Mouth contact frequency (event/h) (n=24)		
Floors	−0.289	0.170
Hands *	−0.631	**0.016**
Non-Dietary	0.378	0.705
Dietary	−0.163	0.448
All objects *	−0.459	**0.024**
Mouth contact duration (min/h) (n = 24)		
Floors	−0.289	0.170
Hands *	−0.593	**0.025**
Non-Dietary *	−0.624	**0.011**
Dietary	−0.095	0.657
All objects *	−0.025	**0.025**
Mouth median contact duration (s)		
Floors (n = 1)	−0.289	0.169
Hands (n = 14)	−0.322	0.261
Non-Dietary * (n = 13)	−0.555	**0.005**
Dietary (n = 6)	−0.122	0.570
All objects * (n = 20)	−0.418	**0.042**

* Significant (p < 0.05) correlation with age (Spearman’s rank correlation).

Table S38. Mouthing contact frequency (event/h) while on playground (n = 24) by EPA age groups.

Age Group		Floors	Hands	Dietary	Non-Dietary	All Objects
< 2<br>N = 5	Mean	0.5	26.6	6.4	27.2	33.6
	SD	1.0	18.6	9.3	14.8	18.9
	Min	0.0	9.2	0.0	5.0	5.0
	p25	0.0	9.2	0.0	24.4	24.4
	Median	0.0	24.4	0.0	30.0	41.5
	p75	0.0	46.2	11.5	30.7	46.2
	p95	2.3	46.2	20.5	46.2	51.1
	p99	2.3	46.2	20.5	46.2	51.1
	Max	2.3	46.2	20.5	46.2	51.1
2 to < 3<br>N = 5	Mean	-	34.4	60.7	47.1	107.8
	SD	-	27.8	135.8	34.4	114.7
	Min	-	7.1	0.0	4.4	16.5
	p25	-	11.7	0.0	16.5	65.2
	Median	-	31.6	0.0	65.2	66.9
	p75	-	57.2	0.0	66.9	82.5
	p95	-	67.5	303.7	82.5	308.1
	p99	-	67.5	303.7	82.5	308.1
	Max	-	67.5	303.7	82.5	308.1
3 to < 6<br>N = 8	Mean	-	10.8	86.5	34.8	121.4
	SD	-	10.3	161.2	74.8	163.2
	Min	-	2.9	0.0	0.0	0.0
	p25	-	3.6	0.0	0.0	1.5
	Median	-	7.3	0.0	6.7	17.9
	p75	-	17.9	156.7	23.5	276.6

	p95	-	25.4	379.0	218.2	379.0
	p99	-	25.4	379.0	218.2	379.0
	Max	-	25.4	379.0	218.2	379.0
6 to < 11 N = 6	Mean	-	4.8	1.1	4.3	5.4
	SD	-	3.9	2.6	4.4	5.0
	Min	-	1.4	0.0	0.0	0.0
	p25	-	1.4	0.0	0.0	0.0
	Median	-	3.9	0.0	3.3	5.9
	p75	-	9.1	0.0	9.1	10.0
	p95	-	9.1	6.4	10.0	10.3
	p99	-	9.1	6.4	10.0	10.3
	Max	-	9.1	6.4	10.0	10.3
p-value		-	0.118	0.833	**0.036**	**0.041**

Table S39. Mouthing hourly duration (min/h) while on playground (n = 24) by EPA age groups.

Age Group		Floors	Hands	Dietary	Non-Dietary	All Objects
< 2 N = 5	Mean	0.0	3.1	0.3	2.7	2.9
	SD	0.0	2.6	0.4	2.0	1.9
	Min	0.0	0.1	0.0	0.3	0.3
	p25	0.0	0.1	0.0	0.8	1.7
	Median	0.0	4.1	0.0	3.1	3.4
	p75	0.0	5.0	0.3	4.1	4.1
	p95	0.0	5.0	1.0	5.0	5.0
	p99	0.0	5.0	1.0	5.0	5.0
	Max	0.05	5.0	1.0	5.0	5.0
2 to < 3 N = 5	Mean	-	0.7	0.9	0.9	1.9
	SD	-	0.7	2.1	0.6	1.7
	Min	-	0.3	0.0	0.2	0.6
	p25	-	0.3	0.0	0.6	1.0
	Median	-	0.5	0.0	1.0	1.1
	p75	-	1.2	0.0	1.1	1.9
	p95	-	1.8	4.6	1.9	4.8
	p99	-	1.8	4.6	1.9	4.8
	Max	-	1.8	4.6	1.9	4.8
3 to < 6 N = 8	Mean	-	0.3	1.8	2.8	4.6
	SD	-	0.2	4.0	7.0	7.6
	Min	-	0.1	0.0	0.0	0.0
	p25	-	0.2	0.0	0.0	0.0
	Median	-	0.3	0.0	0.2	0.4
	p75	-	0.4	1.6	1.0	8.1
	p95	-	0.5	11.4	20.0	20.0
	p99	-	0.5	11.4	20.0	20.0
	Max	-	0.5	11.4	20.0	20.0
6 to < 11 N = 6	Mean	-	0.1	2.8	0.1	2.8
	SD	-	0.1	6.7	0.1	6.7
	Min	-	0.0	0.0	0.0	0.0
	p25	-	0.0	0.0	0.0	0.0
	Median	-	0.1	0.0	0.0	0.0
	p75	-	0.3	0.0	0.1	0.3
	p95	-	0.3	16.5	0.3	16.6

	p99	-	0.3	16.5	0.3	16.6
	Max	-	0.3	16.5	0.3	16.6
p-value		-	0.189	0.961	**0.017**	0.183

Table S40. Mouthing median duration (s) while on playground by EPA age groups.

Age Group		Floors	Hands	Dietary	Non-Dietary	All Objects
< 2	n	1	3	2	4	5
	Mean	1.00	4.50	2.00	4.13	3.50
	SD	-	3.04	1.41	2.59	2.65
	Min	-	1.00	1.00	1.00	1.00
	p25	-	1.00	1.00	2.00	1.00
	Median	-	6.00	2.00	4.50	3.00
	p75	-	6.50	3.00	6.25	6.00
	p95	-	6.50	3.00	6.50	6.50
	p99	-	6.50	3.00	6.50	6.50
	Max	-	6.50	3.00	6.50	6.50
2 to < 3	n	0	4	1	4	5
	Mean	-	1.25	1.00	1.25	1.20
	SD	-	0.50	-	0.50	0.45
	Min	-	1.00	-	1.00	1.00
	p25	-	1.00	-	1.00	1.00
	Median	-	1.00	-	1.00	1.00
	p75	-	1.50	-	1.50	1.00
	p95	-	2.00	-	2.00	2.00
	p99	-	2.00	-	2.00	2.00
	Max	-	2.00	-	2.00	2.00
3 to < 6	n	0	4	2	5	6
	Mean	-	1.75	1.25	2.40	1.92
	SD	-	0.96	1.06	1.67	1.63
	Min	-	1.00	0.50	1.00	0.50
	p25	-	1.00	0.50	1.00	1.00
	Median	-	1.50	1.25	2.00	1.50
	p75	-	2.50	2.00	3.00	2.00
	p95	-	3.00	2.00	5.00	5.00
	p99	-	3.00	2.00	5.00	5.00
	Max	-	3.00	2.00	5.00	5.00
6 to < 11	n	0	3	1	4	4
	Mean	-	1.17	146.00	1.00	30.38
	SD	-	0.29	-	0.82	58.76
	Min	-	1.00	-	0.00	0.00
	p25	-	1.00	-	0.50	0.50
	Median	-	1.00	-	1.00	1.50
	p75	-	1.50	-	1.50	60.25
	p95	-	1.50	-	2.00	118.50
	p99	-	1.50	-	2.00	118.50
	Max	-	1.50	-	2.00	118.50
p-value *		-	0.385	0.431	0.125	0.542

Table S41. Mouthing contact frequency (event/h) while on playground (n = 24) by younger (1–6 years) and older (7–12 years).

Age Group		Floors	Hands	Dietary	Non-	All
1–6 years (n = 21)	Mean	0.11	19.64	49.26	31.75	81.01
	SD	0.50	20.31	119.10	49.09	119.24
	Min	0.00	1.39	0.00	0.00	0.00
	p25	0.00	4.31	0.00	3.85	10.03
	Median	0.00	9.80	0.00	16.45	25.42
	p75	0.00	25.42	6.42	30.68	66.91
	p95	0.00	67.50	313.43	82.50	334.99
	p99	2.30	67.50	378.95	218.18	378.95
	Max	2.30	67.50	378.95	218.18	378.95
7–12 years (n = 3)	Mean	-	-	0.00	3.02	3.02
	SD	-	-	0.00	5.23	5.23
	Min	-	-	0.00	0.00	0.00
	p25	-	-	0.00	0.00	0.00
	Median	-	-	0.00	0.00	0.00
	p75	-	-	0.00	9.05	9.05
	p95	-	-	0.00	9.05	9.05
	p99	-	-	0.00	9.05	9.05
	Max	-	-	0.00	9.05	9.05
p-value		-	-	-	0.072	**0.032**

* Significant ($p < 0.05$). Wilcoxon sum rank test.

Table S42. Mouthing contact duration (min/h) while on playground (n = 24) by younger (1–6 years) and older (7–12 years).

Age Group		Floors	Hands	Dietary	Non-Dietary	All Objects
1–6 years (n = 21)	Mean	0.04	0.98	1.76	1.95	3.71
	SD	0.0	1.59	4.30	4.37	5.70
	Min	0.0	0.02	0.00	0.00	0.00
	p25	0.0	0.12	0.00	0.06	0.33
	Median	0.0	0.28	0.00	0.51	1.08
	p75	0.0	0.67	0.34	1.58	4.14
	p95	0.0	5.00	11.40	5.00	16.58
	p99	0.0	5.00	16.52	20.00	20.00
	Max	0.0	5.00	16.52	20.00	20.00
7–12 years (n = 3)	Mean	-	-	0.00	0.00	0.00
	SD	-	-	0.00	0.00	0.00
	Min	-	-	0.00	0.00	0.00
	p25	-	-	0.00	0.00	0.00
	Median	-	-	0.00	0.00	0.00
	p75	-	-	0.00	0.00	0.00
	p95	-	-	0.00	0.00	0.00
	p99	-	-	0.00	0.00	0.00
	Max	-	-	0.00	0.00	0.00
p-value		-	-	0.301	**0.017**	**0.012**

* Significant ($p < 0.05$). Wilcoxon sum rank test.

Table S43. Mouthing duration (min/h) while children play on playgrounds by gender.

Gender		Floors	Hands	Dietary	Non-Dietary	All objects
Males (n = 11)	Mean	0.04	1.17	0.83	1.10	1.93
	SD	-	1.59	1.58	1.40	1.71
	Min	-	0.05	0.00	0.00	0.00
	p25	-	0.12	0.00	0.00	0.05
	Median	-	0.47	0.00	0.78	1.74
	p75	-	1.75	0.96	1.87	3.41
	p95	-	4.14	4.62	4.14	4.77
	p99	-	4.14	4.62	4.14	4.77
	Max	-	4.14	4.62	4.14	4.77
Females (n = 13)	Mean	-	0.84	2.15	2.21	4.36
	SD	-	1.69	5.35	5.51	7.20
	Min	-	0.02	0.00	0.00	0.00
	p25	-	0.17	0.00	0.04	0.04
	Median	-	0.28	0.00	0.33	0.33
	p75	-	0.42	0.00	0.58	5.00
	p95	-	5.00	16.52	20.00	20.00
	p99	-	5.00	16.52	20.00	20.00
	Max	-	5.00	16.52	20.00	20.00
p-value *		-	0.697	0.402	0.770	0.662

* p-value from Wilcoxon Sum Rank test comparison of male and female

Table S44. Mouthing median duration (s) while children play on playgrounds by gender.

Gender		Floors	Hands	Dietary	Non-Dietary	All objects
Males	n	1	6	4	6	9
	Mean	1.00	1.83	1.38	1.83	1.50
	SD	-	2.04	1.11	2.04	1.70
	Min	-	1.00	0.50	1.00	0.50
	p25	-	1.00	0.75	1.00	1.00
	Median	-	1.00	1.00	1.00	1.00
	p75	-	1.00	2.00	1.00	1.00
	p95	-	6.00	3.00	6.00	6.00
	p99	-	6.00	3.00	6.00	6.00
	Max	-	6.00	3.00	6.00	6.00
Females	n	0	8	2	11	11
	Mean	-	2.25	74.00	2.41	13.00
	SD	-	1.85	101.82	1.91	35.04
	Min	-	1.00	2.00	0.00	0.00
	p25	-	1.00	2.00	1.00	1.00
	Median	-	1.75	74.00	2.00	2.00
	p75	-	2.50	146.00	3.00	5.00
	p95	-	6.50	146.00	6.50	118.50
	p99	-	6.50	146.00	6.50	118.50
	Max	-	6.50	146.00	6.50	118.50
p-value *		-	0.174	0.156	0.264	**0.041**

*** p-value from Wilcoxon Sum Rank test comparison of male and female**

Table S45. Mouthing frequency (event/h) while children play on playgrounds by gender.

Gender		Floors	Hands	Dietary	Non-Dietary	All objects
Males (n = 11)	Mean	2.30	27.87	64.96	27.91	92.87
	SD	-	24.68	137.82	30.70	128.10
	Min	-	2.94	0.00	0.00	0.00
	p25	-	9.22	0.00	0.00	2.94
	Median	-	20.36	0.00	24.43	51.13
	p75	-	46.84	20.45	65.16	82.50
	p95	-	67.50	378.95	82.50	378.95
	p99	-	67.50	378.95	82.50	378.95
	Max	-	67.50	378.95	82.50	378.95
Females (n = 13)	Mean	-	13.46	24.60	28.37	52.97
	SD	-	15.13	86.80	58.44	102.80
	Min	-	1.39	0.00	0.00	0.00
	p25	-	4.08	0.00	3.85	4.96
	Median	-	8.09	0.00	10.03	10.27
	p75	-	17.90	0.00	21.56	25.42
	p95	-	46.15	313.43	218.18	334.99
	p99	-	46.15	313.43	218.18	334.99
	Max	-	46.15	313.43	218.18	334.99
p-value *		-	0.197	0.223	0.705	0.212

* p-value from Wilcoxon Sum Rank test comparison of male and female

Figures

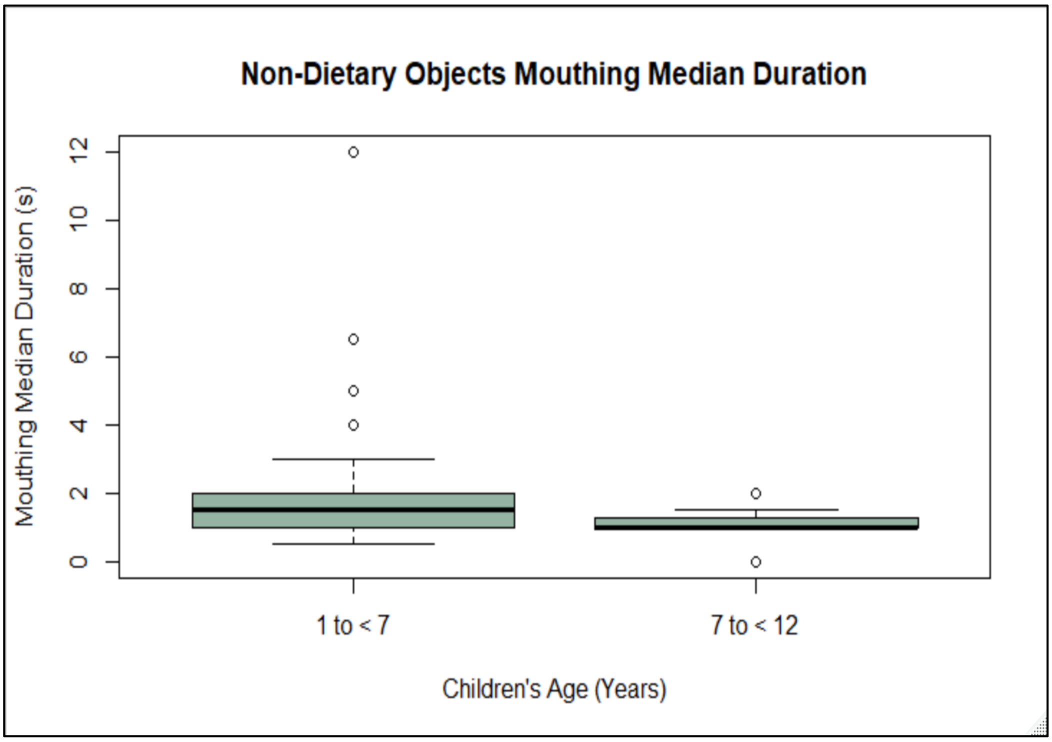

Figure S1. Median mouthing contact duration (s) of younger and older children while playing on turf.
